# Carbon and Nitrogen Isotopic Survey of Northern Peruvian Plants: Baselines for Paleodietary and Paleoecological Studies

**DOI:** 10.1371/journal.pone.0053763

**Published:** 2013-01-16

**Authors:** Paul Szpak, Christine D. White, Fred J. Longstaffe, Jean-François Millaire, Víctor F. Vásquez Sánchez

**Affiliations:** 1 Department of Anthropology, The University of Western Ontario, London, Ontario, Canada; 2 Department of Earth Sciences, The University of Western Ontario, London, Ontario, Canada; 3 Centro de Investigaciones Arqueobiológicas y Paleoecológicas Andinas (ARQUEOBIOS), Trujillo, Peru; New York State Museum, United States of America

## Abstract

The development of isotopic baselines for comparison with paleodietary data is crucial, but often overlooked. We review the factors affecting the carbon (δ^13^C) and nitrogen (δ^15^N) isotopic compositions of plants, with a special focus on the carbon and nitrogen isotopic compositions of twelve different species of cultivated plants (*n* = 91) and 139 wild plant species collected in northern Peru. The cultivated plants were collected from nineteen local markets. The mean δ^13^C value for maize (grain) was −11.8±0.4 ‰ (*n* = 27). Leguminous cultigens (beans, Andean lupin) were characterized by significantly lower δ^15^N values and significantly higher %N than non-leguminous cultigens. Wild plants from thirteen sites were collected in the Moche River Valley area between sea level and ∼4,000 meters above sea level (masl). These sites were associated with mean annual precipitation ranging from 0 to 710 mm. Plants growing at low altitude sites receiving low amounts of precipitation were characterized by higher δ^15^N values than plants growing at higher altitudes and receiving higher amounts of precipitation, although this trend dissipated when altitude was >2,000 masl and MAP was >400 mm. For C_3_ plants, foliar δ^13^C was positively correlated with altitude and precipitation. This suggests that the influence of altitude may overshadow the influence of water availability on foliar δ^13^C values at this scale.

## Introduction

Stable isotope analysis is an important tool for reconstructing the diet, local environmental conditions, migration, and health of prehistoric human and animal populations. This method is useful because the carbon and nitrogen isotopic compositions of consumer tissues are directly related to the carbon and nitrogen isotopic compositions of the foods consumed [1], [2], after accounting for the trophic level enrichments of ^13^C and ^15^N for any particular tissue [3], [4].

In all cases, interpretations of isotopic data depend on a thorough understanding of the range and variation in isotopic compositions of source materials [5]. For instance, studies of animal migrations using oxygen and hydrogen isotopic analyses require a thorough understanding of the spatial variation in surface water and precipitation isotopic compositions [6], and in that avenue of research, there has generally been an emphasis on establishing good baselines. With respect to diet and local environmental conditions, the interpretation of isotopic data (typically the carbon and nitrogen isotopic composition of bone or tooth collagen) depends upon a thorough knowledge of the range and variation in isotopic compositions of foods that may have been consumed. Although several authors have attempted to develop such isotopic baselines for dietary reconstruction [7]–[10], these studies have typically focused on vertebrate fauna.

Despite the fact that plants are known to be characterized by extremely variable carbon and nitrogen isotopic compositions [11], [12], few studies have attempted to systematically document this variability in floral resources at a regional scale using an intensive sampling program, although there are exceptions [13]–[15]. This is problematic, particularly in light of the development and refinement of new techniques (e.g. isotopic analysis of individual amino acids), which will increase the resolution with which isotopic data can be interpreted. If isotopic baselines continue to be given marginal status, the power of new methodological advancements will never be fully realized.

With respect to the Andean region of South America, the isotopic composition of plants is very poorly studied, both from ecological and paleodietary perspectives. The most comprehensive study of the latter type was conducted by Tieszen and Chapman [14] who analyzed the carbon and nitrogen isotopic compositions of plants collected along an altitudinal transect (∼0 to 4,400 masl) following the Lluta River in northern Chile. Ehleringer et al. [16] presented δ^13^C values for plants along a more limited altitudinal transect in Chile (Atacama Desert). A number of other studies have provided isotopic data on a much more limited scale from various sites in Argentina [17]–[21], Chile [22]–[24], Bolivia [25], [26], Ecuador [26], Colombia [26], and Peru [26]–[30].

The number of carbon and nitrogen isotopic studies in the Andean region has increased dramatically in the last ten years, facilitated by outstanding organic preservation in many areas. The majority of these studies have been conducted in Peru [31]–[42] and Argentina [18]–[21], [43]–[47]. With respect to northern Peru in particular, a comparatively small number of isotopic data have been published [40], [48], [49], although this will certainly rise in coming years as biological materials from several understudied polities (e.g. Virú, Moche, Chimú) in the region are subjected to isotopic analysis.

The purpose of this study is to systematically examine the carbon and nitrogen isotopic compositions of plants from the Moche River Valley in northern Peru collected at various altitudes from the coast to the highlands. These data provide a robust baseline for paleodietary, paleoecological, and related investigations in northern Peru that will utilize the carbon and nitrogen isotopic compositions of consumer tissues.

### Study Area

**Figure 1:**
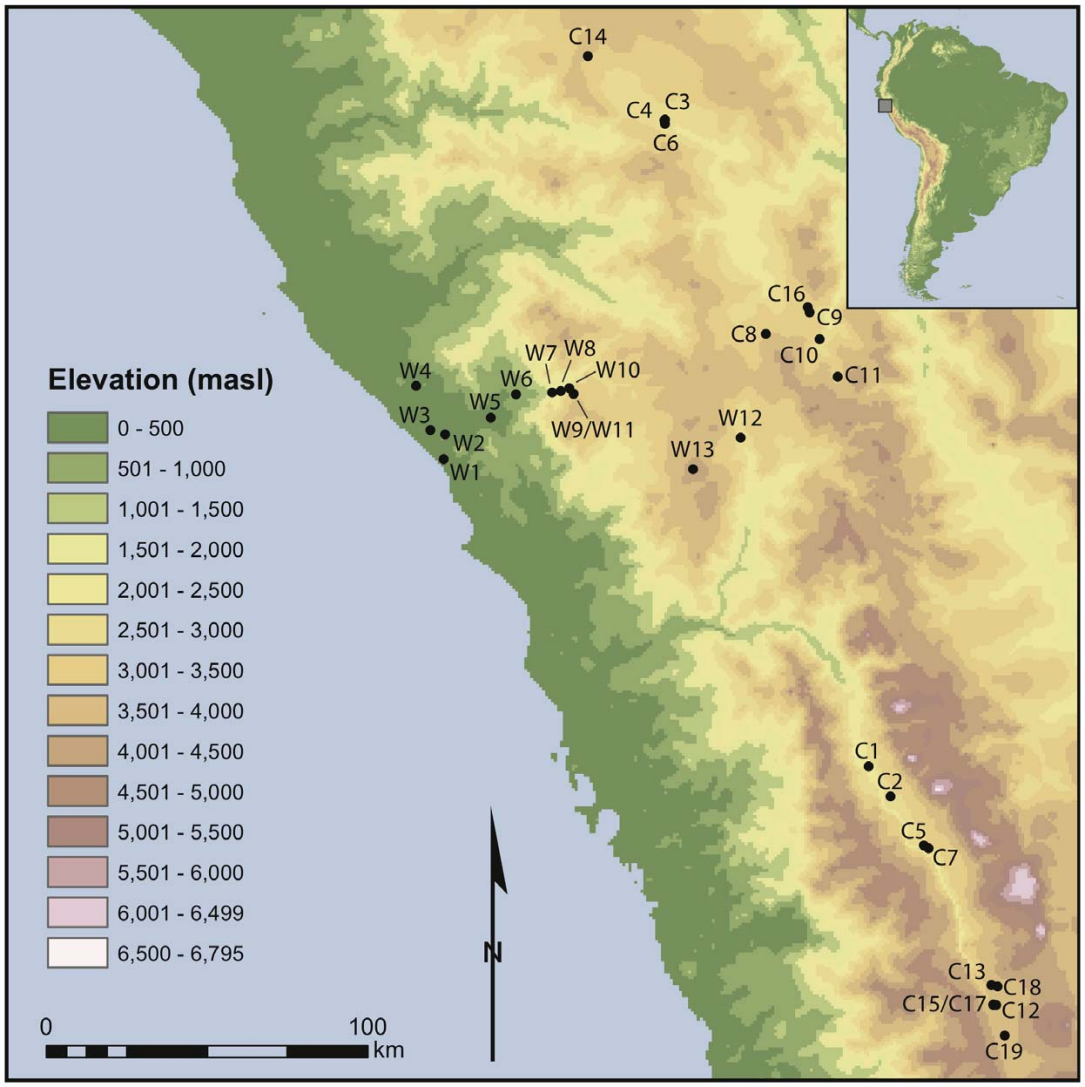
Digital elevation model of the study region derived from the Global 30 Arc-Second Elevation (GTOPO30) data set.

**Figure 2:**
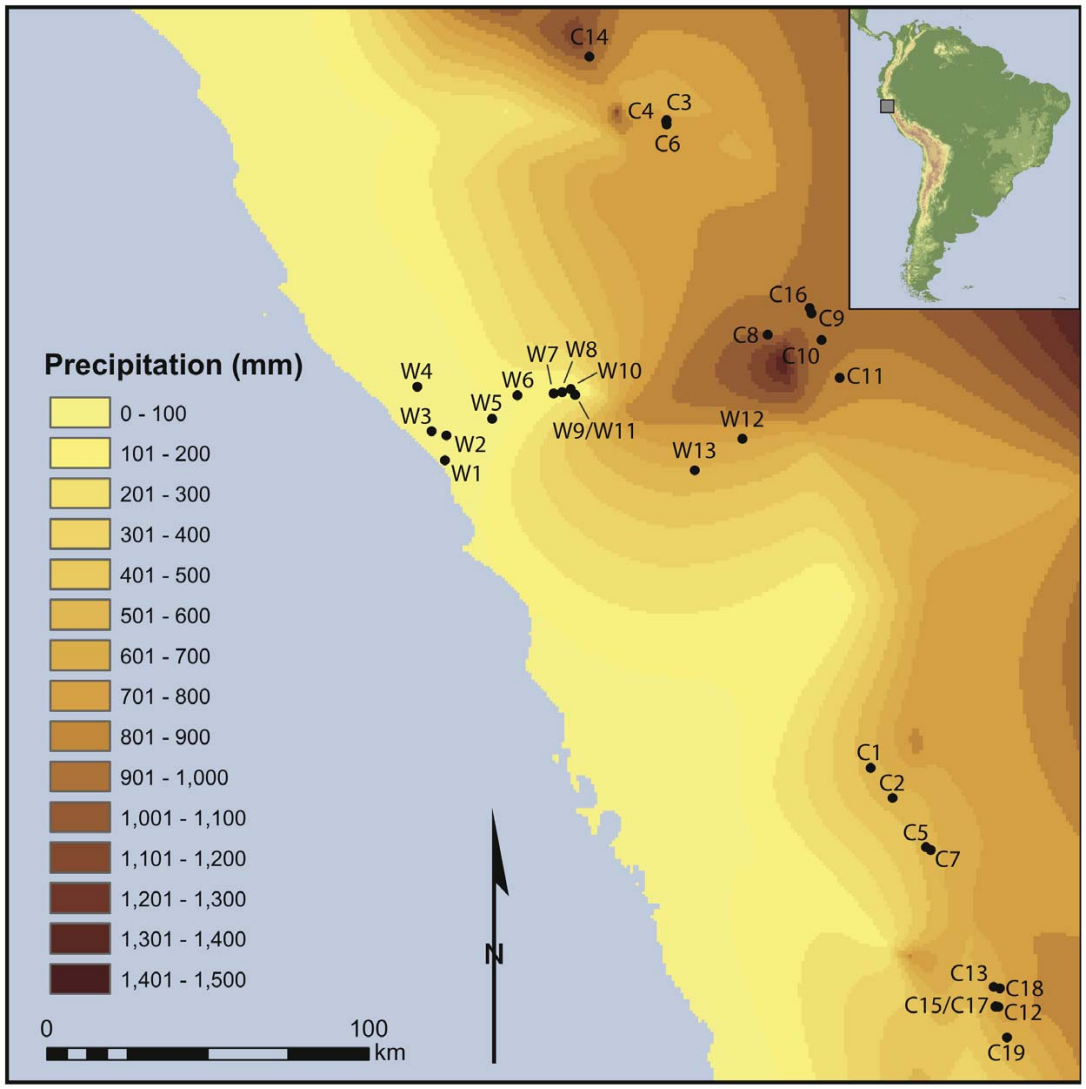
Extrapolated mean annual precipitation for study area. Mean annual precipitation data from 493 monitoring stations in Peru [218] were extrapolated using the natural neighbor method in ArcMap (ArcGIS 10.0, ESRI).

The Andes are an area of marked environmental complexity and diversity. This diversity is driven largely by variation in altitude (Figure 1). As one proceeds from the Pacific coast to the upper limits of the Andes, mean daily temperature declines, typically by ∼5°C per 1,000 m [50], and mean annual precipitation increases (Figure 2). The eastern slope of the Andes, which connects to the Amazon basin, is environmentally very different from the western slope. Because this study deals exclusively with the western slope, the eastern slope is not discussed further. Many authors have addressed the environment of the central Andes [51]–[58], hence only a brief review is necessary here.

The coastal region of Peru is dominated by the hyper-arid Peruvian desert. Cool sea-surface temperatures created by the northward flowing Peruvian Current, combined with a subtropical anticyclone, create remarkably stable and relatively mild temperatures along the roughly 2,000 km north-south extent of the Peruvian desert [55]. The phytogeography of the coastal region of Peru is fairly homogenous, although the composition of the vegetation varies in accordance with local topography [59]. Except in El Niño years, precipitation is extremely low or non-existent along much of the Peruvian coast, but in areas where topography is steep close to the coast, a fog zone forms (typically between 600 and 900 masl), which allows for the development of ephemeral plant communities (*lomas*) [60]–[62]. Aside from these *lomas*, riparian vegetation grows in the relatively lush river valleys that cut into the Andes, although the vast majority of this land is cultivated. Thickets of the leguminous algarroba tree regularly occur at low altitudes, and it is generally believed that much more extensive forests of these trees existed in the past [63], [64]. The coastal zone usually ends where the oceanic influence becomes minimal, typically about 1,000 masl [58].

Immediately above the area of oceanic influence and up to an altitude of ∼1,800 m, the environment is cooler, although generally similar, in comparison to the coastal zone. Although mean annual precipitation increases, this zone can still be characterized as dry, with most locations receiving less than 400 mm of annual precipitation. In some circumstances, *lomas* may form within this zone [52], although this is not common. In the Moche River Valley of northern Peru, the vegetation is dominated by xerophytic scrub vegetation from 500 to 1,800 masl, and transitions to thorny steppe vegetation between 1,800 and 2,800 masl. Again, the area is still characterized by relatively low annual precipitation, although water availability is greater close to major watercourses and other ground water sources. Ascending further, mean annual precipitation increases, and average daily temperature decreases. Night frost begins to occur. Vegetation is largely dominated by low-growing shrubs, herbs, and grasses, as well as open stands of some tree species (*Acacia*, *Polylepis*) [56]. Pastures dominated by dense bunchgrasses occur in moister areas.

### Natural Variation in Plant Carbon Isotopic Composition

#### Photosynthetic pathway and taxonomy

The most salient mechanism influencing the carbon isotopic composition (δ^13^C) of terrestrial plants is the photosynthetic pathway utilized. Plants that fix carbon using the C_3_ pathway (Calvin cycle) are characterized by lower δ^13^C values (ca. −26 ‰) than plants utilizing the C_4_ (Hatch-Slack) pathway (ca. −12 ‰) [65], [66]. This is because carbon isotope discrimination (Δ^13^C) is smaller in C_4_ plants than in C_3_ plants. In other words, C_3_ plants discriminate more strongly against the heavier isotope (^13^C) than C_4_ plants. The vast majority of C_4_ plants are tropical grasses, the most significant of which in New World archaeological contexts is maize (*Zea mays*), but also amaranth (*Amaranthus caudatus*). With respect to human diet, most wild C_4_ plants are not significant, and thus a large body of research has focused on assessing and quantifying the contribution of C_4_ cultigens (mostly maize, but also millet) to the diet [67]. Some desert plants and succulents exhibit carbon isotopic compositions that are intermediate between C_3_ and C_4_ plants. Referred to as CAM (Crassulacean acid metabolism) plants, these species fix carbon in a manner analogous to C_4_ plants overnight, but utilize the C_3_ photosynthetic pathway during the afternoon [68].

Additional plant groups that are not readily assigned into the aforementioned categories include mosses and lichens. Mosses, which are non-vascular plants, utilize the C_3_ photosynthetic pathway [69], [70], but are distinct from vascular plants in that they lack stomata and CO_2_ availability is influenced primarily by the thickness of the water film accumulated on the leaves. Lichens are composite organisms, consisting of two parts: a mycobiont (fungi) and photobiont or phycobiont (algae). The carbon isotopic composition of lichens is determined largely by the type of photobiont involved. Lichens with green algae as the photobiont exhibit a wide range of carbon isotopic compositions (−35 to −17 ‰), while lichens with cyanobacteria as the photobiont tend to have higher, and a more restricted range of carbon isotopic compositions (−23 to −14 ‰) [71]–[73].

#### Environmental factors affecting plant δ^13^C

Aside from the differences in carbon isotopic composition resulting from variable carbon fixation, a number of environmental factors have also been demonstrated to influence the carbon isotopic composition of plant tissues. For example, low-growing plants under dense forest cover tend to exhibit lower δ^13^C values relative to canopy plants and plants growing in more open environments. Often referred to as the ‘canopy effect’, this is attributed to relatively ^13^C-depleted CO_2_ in the understory due to the utilization of recycled CO_2_ [74]–[78], and/or lower irradiance and higher [CO_2_] relative to the canopy [79], [80]. The magnitude of differences in plant carbon isotopic composition observed due to the canopy effect typically range between 2 and 5 ‰ [81]. It has been posited that the canopy effect significantly impacts the carbon isotopic composition of consumer tissues and thus reflects the use of closed and open habitats [82]–[84]. None of the sites sampled in this study were characterized by sufficiently dense forest for a canopy effect to have been significant.

Water availability has been observed to be negatively correlated with the carbon isotopic composition of plants [85]–[91]. In most instances, these effects are limited to C_3_ plants, with most studies finding little or no correlation between rainfall and/or water availability and plant δ^13^C for C_4_ plants [86], [92]. Murphy and Bowman [87] found a positive correlation between rainfall and C_4_ plant δ^13^C over a continental (Australia) rainfall gradient, although this relationship is atypical. It is believed that the relationship between aridity and plant δ^13^C is caused by increased stomatal closure when water availability is low, which is accompanied by decreased discrimination against ^13^C during photosynthesis and, in turn, comparatively less negative δ^13^C values [93], [94].

Soil salinity has also been demonstrated to influence plant δ^13^C values. In a manner somewhat analogous to drought stress, salt stress induces increased stomatal closure, and therefore reduces discrimination against ^13^C by the plant [95]. A number of studies have observed this relationship, which occurs in both halophytic (salt-tolerant) [96], [97] and non-halophytic species [98], [99].

A number of studies have found elevational gradients in plant carbon isotopic composition. Generally, foliar δ^13^C values have been found to increase with increasing altitude [88], [100], [101]. It is important to point out, however, that the majority of these studies have examined the isotopic composition of a single species or a small number of species over an elevational gradient of ∼1,000 m. The exact mechanism responsible for the relationship between plant δ^13^C and altitude is not entirely clear. Some have suggested exceptionally high carboxylation rates relative to stomatal conductance [102], [103] and/or high carboxylation efficiency [104] for plants growing at high altitudes, resulting in decreased discrimination against ^13^C. A very strong positive correlation has been observed between altitude and leaf mass per unit area [100], [101], which is thought to be instrumental in increasing carboxylation capacity.

Irradiance has also been shown to influence foliar δ^13^C values, with higher irradiance being associated with less negative δ^13^C values in leaves. Such variation can occur within a single plant (usually trees), and even along a single branch, with leaves growing in interior, shaded areas having lower δ^13^C values than leaves growing in exterior, exposed areas [105], [106]. These differences in δ^13^C associated with irradiance have been attributed to differences in intercellular CO_2_ concentration [94].

#### Intraplant and temporal variation in plant δ^13^C

Carbon isotopic composition is not necessarily equal among different plant parts. Numerous studies have observed variation in the δ^13^C values of leaves, stems, roots, and other tissues [107]–[109]. The vast majority of studies examining the carbon isotopic compositions of multiple plant tissues have found that leaves are slightly depleted of ^13^C relative to non-photosynthetic tissues, typically by 2 to 4 ‰ [108], [110], [111]. These differences are only consistent among C_3_ plants, with C_4_ plants often showing little variation between leaves and non-photosynthetic tissues, or leaves with relatively high δ^13^C values in some cases [107], [108]. There are several potential variables contributing to intraplant variation in tissue δ^13^C. First, different tissues may contain variable proportions of molecules that are relatively enriched or depleted of ^13^C compared to total organic matter. Most notably, lipids [112] and lignin [113] are known to be characterized by relatively low δ^13^C values, while the opposite is true for cellulose, sugars, and starches [114]. Because some studies have found significant differences in the δ^13^C of specific compounds (e.g. cellulose, sucrose) between different plant parts [110], [111], it is thought that additional mechanisms are responsible for the observed patterns in intraplant δ^13^C variation. Damesin and Lelarge [110] suggest that some discrimination occurs during the translocation of sugars, particularly when certain plasma membrane proteins are involved in phloem transport. Potential mechanisms causing intraplant variation in δ^13^C are treated at length by Cernusak et al. [109].

In addition to variation among plant parts, a number of studies have found variation in δ^13^C within plant parts, over time. Specifically, emerging leaves, which are not yet photosynthetic and therefore more closely resemble other non-photosynthetic or heterotrophic plants parts, tend to have less negative δ^13^C values (by about 1 to 3 ‰) relative to fully emerged, photosynthetic leaves [91], [110], [111]. Products assimilated via photosynthesis will tend to have lower δ^13^C values than those acquired heterotrophically, and this is likely partly responsible for the decrease in leaf δ^13^C over time [115].

#### Marine plants

For the purpose of this paper, ‘marine plants’ refers specifically to macroalgae, or plants that are typically classified as kelps, seaweeds, and seagrasses. One of the most commonly reported distinctions in carbon isotopic composition is that marine animals tend to have higher δ^13^C values than terrestrial animals, except in cases where C_4_ plants dominate the diet of the latter. While this distinction holds in the vast majority of circumstances [8], [116], [117], the same relationship is not necessarily true for marine and terrestrial plants.

**Figure 3:**
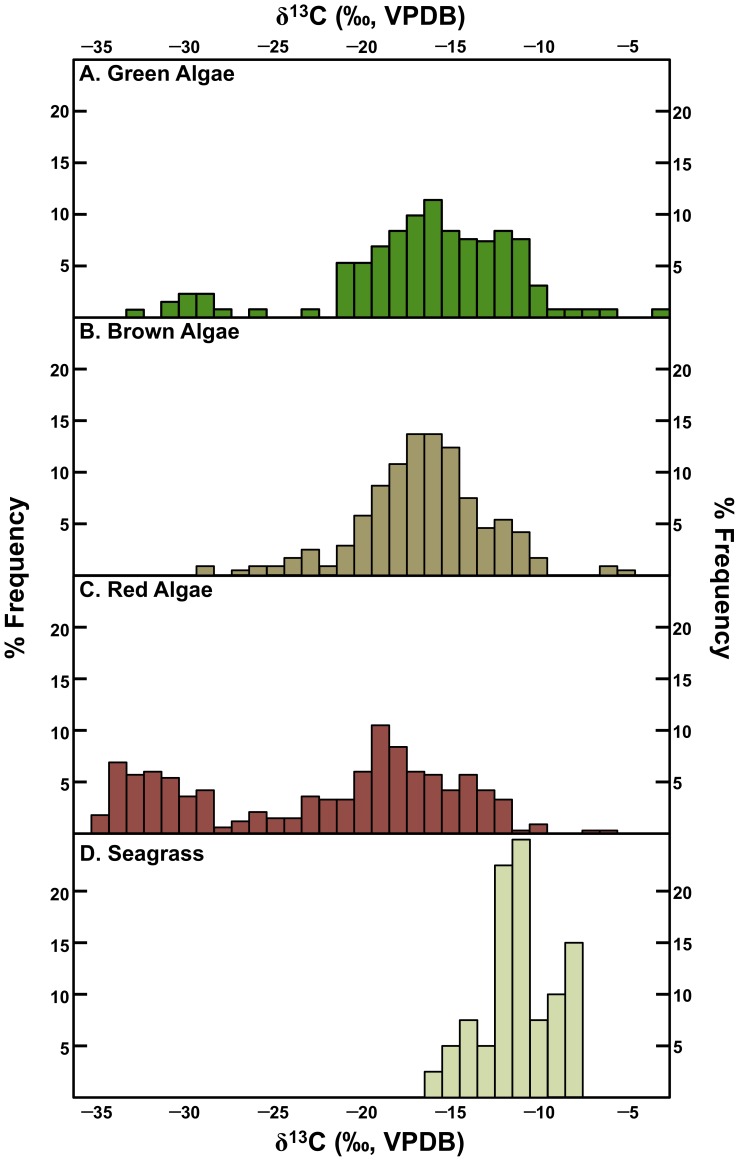
Frequency distributions of carbon isotopic compositions of marine macroalgae. Data are taken from published literature [119], [219]–[235].

Marine plants are characterized by a high degree of variability in carbon isotopic composition. Figure 3 presents the carbon isotopic compositions for the four major classes of marine macroalgae. In general, marine plants are characterized by carbon isotopic compositions that are intermediate in comparison to terrestrial C_3_ and C_4_ plants, with two notable exceptions. Seagrasses (*Zostera* sp.), have extremely high δ^13^C values, typically higher than most terrestrial C_4_ plants (Figure 3d). There is evidence to suggest C_4_ photosynthetic activity in a few species of marine algae [118], but the comparatively high δ^13^C values observed in many species, including seagrasses, cannot typically be explained in this way [119]. The variable use of dissolved CO_2(aq)_ and HCO_3_^−^_(aq)_ is a significant factor, as δ^13^C of HCO_3_^−^_(aq)_ is ∼9 ‰ less negative than that of CO_2(aq)_ [120]. Moreover, for intertidal plants, which are exposed to the atmosphere for a portion of the day, the utilization of atmospheric CO_2_ further complicates matters [119]. The thickness of the diffusive boundary layer is also a potentially important factor with respect to Δ^13^C as it may differ due to variable water velocity [121], [122]. Other environmental factors have also been demonstrated to influence aquatic plant δ^13^C values, such as: salinity [123], extracellular CO_2_ concentration [124], [125], light intensity [123], algal growth rate [126], water velocity [122], and water temperature [127].

Some red algae (Floridiophyceae) are characterized by consistently very low δ^13^C values (<−30 ‰). In general, the brown algae (kelps) have been noted to contribute significantly to nearshore ecosystems in terms of secondary production, with numerous studies examining the relative contributions of offshore phytoplankton and nearshore macroalgae [128].

### Natural Variation in Plant Nitrogen Isotopic Composition

#### Nitrogen Source

Unlike carbon, which is obtained by plants as atmospheric CO_2_, nitrogen is actively taken up from the soil in the vast majority of cases. The two most important nitrogenous species utilized by plants are nitrate (NO_3_^−^) and ammonium (NH_4_^+^). In general, nitrate is the most abundant form of mineralized nitrogen available to plants, but in some instances, such as waterlogged or acidic soils, ammonium may predominate [129], [130]. Additionally, some plants rely, at least to some extent, on atmospheric nitrogen (N_2_), which is obtained by symbiotic bacteria residing in root nodules (rhizobia) [131]. Plants may also take up organic nitrogen (e.g. free amino acids) from the soil [132], although the relative importance of such processes is not well understood and relatively poorly documented [133], [134]. The extent to which plants rely on these N sources is significant because they may have distinct nitrogen isotopic compositions due to fractionations associated with different steps in the nitrogen cycle (e.g. ammonification, nitrification, denitrification), as well as the uptake and eventual incorporation of mineralized N into organic N [135]–[137].

There are two important aspects of variation in N source pertinent to the present study. The first relates to N_2_-fixation by plants (mostly members of Fabaceae), which are common in both wild and domestic contexts in many parts of the central Andes. Plants that utilize significant amounts of atmospheric N_2_ are characterized by comparatively low δ^15^N values, typically ∼0 ‰ [27], [138]–[140]. These plants acquire such compositions because the δ^15^N of atmospheric N_2_ is ∼0 ‰ [141] and the assimilation of N from N_2_-fixation is not associated with significant fractionation of ^15^N [138]–[140]. By comparison, soil NO_3_^−^ and NH_4_^+^ tend to have δ^15^N values >0 ‰ [142], and non N_2_-fixing plants have δ^15^N values that tend to be >0 ‰, although these are highly variable for a number of reasons as discussed in more detail below.

The second potentially significant source-related cause of plant δ^15^N variation is the uptake of fertilizer-derived N by plants. Animal fertilizers are characterized by extremely variable δ^15^N values depending on the relative proportions of N-bearing species in the fertilizer (e.g. urea, uric acid, ammonium, organic matter) [143]. Manures consisting primarily of solid waste derived from terrestrial herbivores tend to have δ^15^N values between 2 and 8 ‰ [144], while those that contain a mix of solid and liquid waste (slurry fertilizers) tend to have higher δ^15^N values, often between 6 and 15 ‰ [145], [146]. The highest δ^15^N values for animal fertilizers (>25 ‰) have been recorded for seabird guano [143], [147], which consists primarily of uric acid and is subject to significant NH_4_^+^ volatilization. The addition of animal fertilizer N to the soil therefore adds an N source with an isotopic composition that is usually enriched in ^15^N relative to endogenous soil N. This results in higher δ^15^N values for plants growing in soils fertilized with animal waste than those plants growing in unfertilized soil or soils fertilized with chemical fertilizers [143], [145]–[147].

Animal-derived N may be delivered to plants by means other than purposeful application of manures. Several studies have documented that the addition of N from animal carcasses (salmon in particular) provide substantial quantities of N taken up by plants. These plants tend to be characterized by relatively high δ^15^N values [148], [149]. Increased grazing intensity has also been suggested to influence plant δ^15^N values due to the concentrated addition of animal waste, but studies have produced conflicting results, with some finding grazing to: increase plant δ^15^N values [150], [151], decrease plant δ^15^N values [152], [153], have little or no impact on plant δ^15^N values [154], [155], or increase δ^15^N in plant roots, but decrease δ^15^N in shoots [156].

#### Taxonomic variation

Strong distinctions in plant δ^15^N have been related to mycorrhizal (fungal) associations [12], [157], [158]. In some ecosystems, particularly those at high latitudes characterized by soils with low N content, this facilitates the distinction between plant functional types – trees, shrubs, and grasses [159]–[161]. In a global survey of foliar δ^15^N values, Craine et al. [12] found significant differences in plant δ^15^N on the basis of mycorrhizal associations, with the following patterns (numbers in parentheses are differences relative to non-mycorrhizal plants): ericoid (−2 ‰), ectomycorrhizal (−3.2 ‰), arbuscular (−5.9 ‰). The comparatively low δ^15^N values of plants with mycorrhizal associations has been attributed to a fractionation of 8 to 10 ‰ against ^15^N during the transfer of N from fungi to plants [162], [163], with the lowest values indicating higher retention of N in the fungi compared to the plant [164].

#### Intraplant and temporal variation in plant δ^15^N

There are three main reasons that plants exhibit intraplant and temporal variation in their tissue δ^15^N values: (1) fractionations associated with NO_3_^−^ assimilation in the root vs. shoot, (2) movement of nitrogenous compounds between nitrogen sources and sinks, (3) reliance on isotopically variable N sources as tissue forms over time.

Both NO_3_^−^ and NH_4_^+^ are taken up by plant roots. NO_3_^−^ can be immediately assimilated into organic N in the root, or it may be routed to the shoot and assimilated there. The assimilation of NO_3_^−^ into organic N is associated with a fractionation of ^15^N of up to −20 ‰ [137], [165]. Therefore, the NO_3_^−^ that is moved to the shoot has already been exposed to some fractionation associated with assimilation and is enriched in ^15^N compared to the NO_3_^−^ that was assimilated in the root. On this basis, it is expected that shoots will have higher δ^15^N values than roots in plants fed with NO_3_^−^ [166]. Because NH_4_^+^ is assimilated only in the root, plants with NH_4_^+^ as their primary N source are not expected to have significant root/shoot variation in δ^15^N [136].

As plants grow they accumulate N in certain tissues (sources) and, over time, move this N to other tissues (sinks). In many species, annuals in particular, large portions of the plant’s resources are allocated to grain production or flowering. In these cases, significant portions of leaf and/or stem N is mobilized and allocated to the fruits, grains, or flowers [167]. When stored proteins are hydrolyzed, moved, and synthesized, isotopic fractionations occur [168], [169]. Theoretically, nitrogen sources (leaves, stems) should be comparatively enriched in ^15^N in relation to sinks (grains, flowers), which has been observed in several studies [143], [145], [147].

In agricultural settings, the variation within a plant over time may become particularly complex due to the application of nitrogenous fertilizers. The availability of different N-bearing species from the fertilizer (NH_4_^+^, NO_3_^−^) and the nitrogen isotopic composition of fertilizer-derived N changes over time as various soil processes (e.g. ammonification, nitrification) occur. The nature of this variation is complex and will depend on the type of fertilizer applied [147].

#### Environmental factors affecting plant **δ^15^N**

Plant nitrogen isotopic compositions are strongly influenced by a series of environmental factors. The environmental variation in plant δ^15^N can be passed on to consumers and cause significant spatial variation in animal isotopic compositions at regional and continental scales [170]–[175].

Plant δ^15^N values have been observed to be positively correlated with mean annual temperature (MAT) [176], [177], although this relationship appears to be absent in areas where MAT ≤ −0.5°C [12]. A large number of studies have found a negative correlation between plant δ^15^N values and local precipitation and/or water availability. These effects have been demonstrated at regional or continental [15], [85]–[87], [172], [178], and global [12], [176], [179] scales. Several authors have hypothesized that relatively high δ^15^N values in herbivore tissues may be the product of physiological processes within the animal related to drought stress [171], [173], [174], although controlled experiments have failed to provide any evidence supporting this hypothesis [180]. More recent research has demonstrated a clear link between herbivore tissue δ^15^N values and plant δ^15^N values, while providing no support for the ‘physiological stress hypothesis’ [172], [181].

The nature of the relationship between rainfall and plant δ^15^N values appears to be extremely complex, with numerous variables contributing to the pattern. Several authors, including Handley et al. [179], have attributed this pattern to the relative ‘openness’ of the nitrogen cycle. In comparison to hot and dry systems, which are prone to losses of excess N, colder and wetter systems more efficiently conserve and recycle mineral N [176] and are thus considered less open. With respect to ecosystem δ^15^N, ^15^N enrichment will be favored for any process that increases the flux of organic matter to mineral N, or decreases the flux of mineral N into organic matter [178]. For instance, low microbial activity, or high NH_3_ volatilization would cause an overall enrichment in ^15^N of the soil-plant system.

#### Marine plants

In comparison to terrestrial plants, the factors affecting the nitrogen isotopic composition of marine plants have not been investigated intensively other than the influence of anthropogenic nitrogen. As is the case with terrestrial plants, marine plant δ^15^N values are strongly influenced by the forms and isotopic composition of available N [182], [183]. Specifically, the relative reliance on upwelled NO_3_^−^ relative to recycled NH_4_^+^ will strongly influence the δ^15^N of marine producers, including macroalgae. Systems that are nutrient poor (oligotrophic) tend to be more dependent on recycled NH_4_^+^, and systems that are nutrient rich (eutrophic) tend to be more dependent on upwelled NO_3_^−^. This results in nutrient-rich, upwelling systems being enriched in ^15^N relative to oligotrophic systems [184].

## Materials and Methods

### Sample Collection

**Figure 4:**
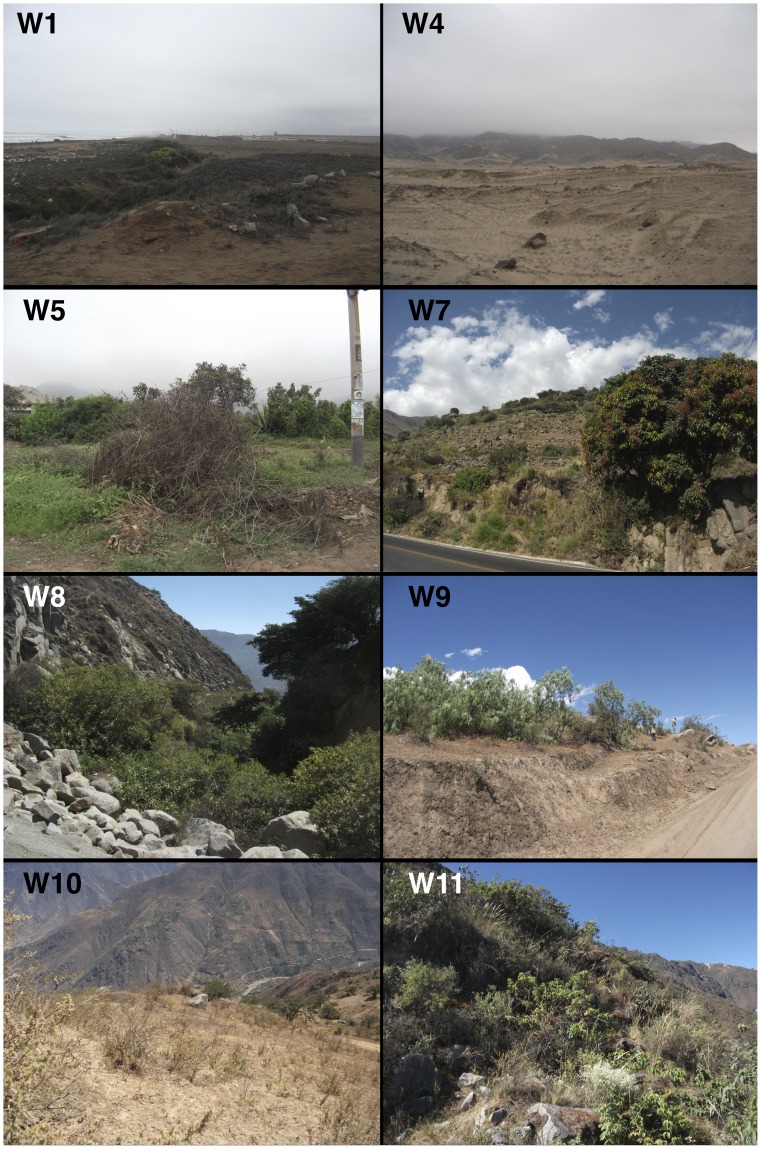
Images of eight of the wild plant sampling locations. Corresponding geographical data for these sites can be found in Table 6.

Wild plants were collected between 2011/07/18 and 2011/08/03. We used regional ecological classifications defined by Tosi [54], which are summarized in Table 1. In each of these five zones, two sites were selected that typified the composition of local vegetation. Sampling locations were chosen to minimize the possibility of significant anthropogenic inputs; in particular, areas close to agricultural fields and disturbed areas were avoided. Sampling locations were fairly open and did not have significant canopy cover. At each sampling location, all plant taxa within a 10 m radius were sampled. Wherever possible, three individuals of each species were sampled and were later homogenized into a single sample for isotopic analysis. Images for eight of the wild plant sampling locations are presented in Figure 4.

**Table 1:** Ecological zones used for sampling in this study [54].

Zone	Altitude
Coastal desert	0 − 500 masl
Premontane desert scrub	500 − 1,800 masl
Premontane thorny steppe	1,800 − 2,800 masl
Montane moist pasture	2,800 − 3,700 masl
Montane wet pasture	3,700 − 4,200 masl

Cultigens (edible portions) were collected from local markets between 2008/10/08 and 2008/11/09 (Table 2). Plants introduced to the Americas were not collected (e.g. peas, barley), even though these species were common. Entire large cultigens (e.g. tubers) were selected and subsequently, a thin (ca. 0.5 cm) slice was sampled. For smaller cultigens (e.g. maize, beans, quinoa) one handful of material was sampled.

**Table 2:** Environmental data for market plant sampling sites.

Site ID	Site Name	Latitude	Longitude	Altitude (masl)
C1	Caraz	−9.0554	−77.8101	2233
C2	Yungay	−9.1394	−77.7481	2468
C3	Jesus	−7.2448	−78.3797	2530
C4	Jesus II	−7.2474	−78.3821	2573
C5	Ampu	−9.2757	−77.6558	2613
C6	Shuto	−7.2568	−78.3807	2629
C7	Carhuaz	−9.2844	−77.6422	2685
C8	Yamobamba	−7.8432	−78.0956	3176
C9	Huamachuco	−7.7846	−77.9748	3196
C10	Curgos	−7.8599	−77.9475	3220
C11	Poc Poc	−7.9651	−77.8964	3355
C12	Recuay	−9.7225	−77.4531	3400
C13	Olleros	−9.6667	−77.4657	3437
C14	Hierba Buena	−7.0683	−78.5959	3453
C15	Mirador II	−9.7220	−77.4601	3466
C16	Yanac	−7.7704	−77.9799	3471
C17	Mirador I	−9.7224	−77.4601	3477
C18	Conray Chico	−9.6705	−77.4484	3530
C19	Catac	−9.8083	−77.4282	3588

For both wild plants and cultigens, geospatial data were recorded using a Garmin® Oregon® 450 portable GPS unit (Garmin®, Olathe, KS, USA). After collection, plants were air-dried on site. Prior to shipping, plants were dried with a Salton® DH−1171 food dehydrator (Salton Canada, Dollard-des-Ormeaux, QC, Canada). Plants were separated according to tissue (leaf, stem, seed, flower). For grasses, all aboveground tissues were considered to be leaf except where significant stem development was present, in which case, leaf and stem were differentiated. All geospatial data associated with these sampling sites are available as a Google Earth.kmz file in the Supporting Information (Dataset S1).

Plants were not sampled from privately-held land or from protected areas. Endangered or protected species were not sampled. Plant materials were imported under permit #2011−03853 from the Canadian Food Inspection Agency. No additional specific permissions were required for these activities.

### Sample Preparation

Samples were prepared according to Szpak et al. [143] with minor modifications. As described above, plant material was dried prior to arrival in the laboratory. Whole plant samples were first homogenized using a Magic Bullet® compact blender (Homeland Housewares, Los Angeles, CA, USA). Ground material was then sieved, with the <180 µm material retained for analysis in glass vials. If insufficient material was produced after sieving, the remaining material was further ground using a Wig-L-Bug mechanical shaker (Crescent, Lyons, IL, USA) and retained for analysis in glass vials. Glass vials containing the ground material were dried at 90°C for at least 48 h under normal atmosphere.

### Stable Isotope Analysis

Isotopic (δ^13^C and δ^15^N) and elemental compositions (%C and %N) were determined using a Delta V isotope ratio mass spectrometer (Thermo Scientific, Bremen, Germany) coupled to an elemental analyzer (Costech Analytical Technologies, Valencia, CA, USA), located in the Laboratory for Stable Isotope Science (LSIS) at the University of Western Ontario (London, ON, Canada). For samples with <2% N, nitrogen isotopic compositions were determined separately, with excess CO_2_ being removed with a Carbo-Sorb trap (Elemental Microanalysis, Okehampton, Devon, UK) prior to isotopic analysis.

Sample δ^13^C and δ^15^N values were calibrated to VPDB and AIR, respectively, with USGS40 (accepted values: δ^13^C = −26.39 ‰, δ^15^N = −4.52 ‰) and USGS41 (accepted values: δ^13^C = 37.63 ‰, δ^15^N = 47.6 ‰). In addition to USGS40 and USGS41, internal (keratin) and international (IAEA-CH-6, IAEA-N-2) standard reference materials were analyzed to monitor analytical precision and accuracy. A δ^13^C value of −24.03±0.14 ‰ was obtained for 81 analyses of the internal keratin standard, which compared well with its average value of −24.04 ‰. A δ^13^C value of −10.46±0.09 ‰ was obtained for 46 analyses of IAEA-CH-6, which compared well with its accepted value of −10.45 ‰. Sample reproducibility was ±0.10 ‰ for δ^13^C and ±0.50% for %C (50 replicates). A δ^15^N value of 6.37±0.13 ‰ was obtained for 172 analyses of an internal keratin standard, which compared well with its average value of 6.36 ‰. A δ^15^N value of 20.3±0.4 ‰ was obtained for 76 analyses of IAEA-N-2, which compared well with its accepted value of 20.3 ‰. Sample reproducibility was ±0.14 ‰ for δ^15^N and ±0.10% for %N (84 replicates).

### Data Treatment and Statistical Analyses

Plants were grouped into the following major functional categories for analysis: herb/shrub, tree, grass/sedge, vine. Plants that are invasive and/or introduced species were included in the calculation of means for particular sites since their isotopic compositions should still be impacted by the same environmental factors as other plants. For all statistical analyses of carbon isotopic composition, grass/sedge and herb/shrub were further separated into C_3_ and C_4_ categories. For comparisons among plant functional types, and sampling sites, foliar tissue was used since other tissues were not as extensively sampled.

Correlations between foliar isotopic compositions and environmental parameters (altitude, mean annual precipitation) were assessed using Spearman’s rank correlation coefficient (*ρ*). One-way analysis of variance (ANOVA) followed by either a Tukey’s HSD test (if variance was homoscedastic) or a Dunnett’s T3 test (if variance was not homoscedastic) was used to compare means. All statistical analyses and regressions were performed in SPSS 16 for Windows.

## Results

### Cultigens

**Figure 5:**
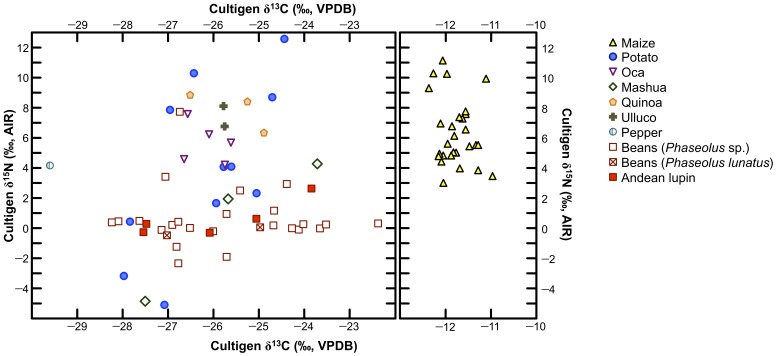
Carbon and nitrogen isotopic compositions of cultigens. Note that the x-axis is not continuous.

The carbon and nitrogen isotopic compositions were analyzed for a total of 85 cultigen samples from eleven species. Carbon and nitrogen isotopic compositions for cultigens are presented in Figure 5. Mean δ^13^C and δ^15^N values for cultigens are presented in Table 3. Isotopic and elemental data, as well as corresponding geospatial data for individual cultigens are presented in Table S1. All isotopic and elemental compositions for cultigens are for consumable portions of the plant, with one exception (maize leaves), which is excluded from Table 3 and Figure 5. Mean δ^13^C values for C_3_ cultigens ranged from −29.8±0.9 ‰ (coca) to −25.6±1.9 ‰ (mashua). The mean δ^13^C value for maize, which was the only C_4_ plant examined, was −11.8±0.4 ‰. Mean δ^15^N values for cultigens were typically more variable than δ^13^C values, ranging from −0.2±0.4 ‰ (*Phaseolus lunatus*) to 7.9±1.3 ‰ (quinoa).

**Table 3:** Mean carbon and nitrogen isotopic compositions for cultigens (±1σ).

Common Name	Taxonomic Name	*n*	δ^13^C (‰, VPDB)	δ^15^N (‰, AIR)	%C	%N
Beans	*Phaseolus* sp.	24	−25.7±1.6	0.7±2.0	39.8±0.7	3.7±0.6
Beans (Lima)	*Phaseolus lunatus*	2	−26.0±1.4	−0.2±0.4	39.0±0.3	2.7±0.5
Chocho (Andean lupin)	*Lupinus mutabilis*	5	−26.0±1.6	0.6±1.2	48.3±2.8	6.8±1.3
Coca	*Erythroxylum coca*	4	−29.8±0.9	−	45.4±1.5	−
Maize (Grain)	*Zea mays*	27	−11.8±0.4	6.4±2.2	40.4±0.5	1.2±0.2
Maize (Leaf)	*Zea mays*	2	−12.9±0.4	4.5±1.6	41.9±4.6	1.3±1.3
Mashua	*Tropaeolum tuberosum*	3	−25.6±1.9	0.5±4.7	41.5±2.8	3.0±0.7
Oca	*Oxalis tuberosa*	6	−26.4±0.7	5.7±1.3	43.1±3.2	1.6±0.6
Pepper	*Capsicum annuum*	1	−29.6	4.2	48.3	2.1
Potato	*Solanum tuberosum*	12	−26.3±1.3	4.0±5.5	40.5±1.5	1.4±0.4
Quinoa	*Chenopodium quinoa*	3	−25.6±0.9	7.9±1.3	39.9±2.1	2.6±0.3
Ulluco	*Ullucus tuberosus*	2	−25.8±0.0	7.5±1.0	40.6±0.4	3.4±1.0

**Figure 6:**
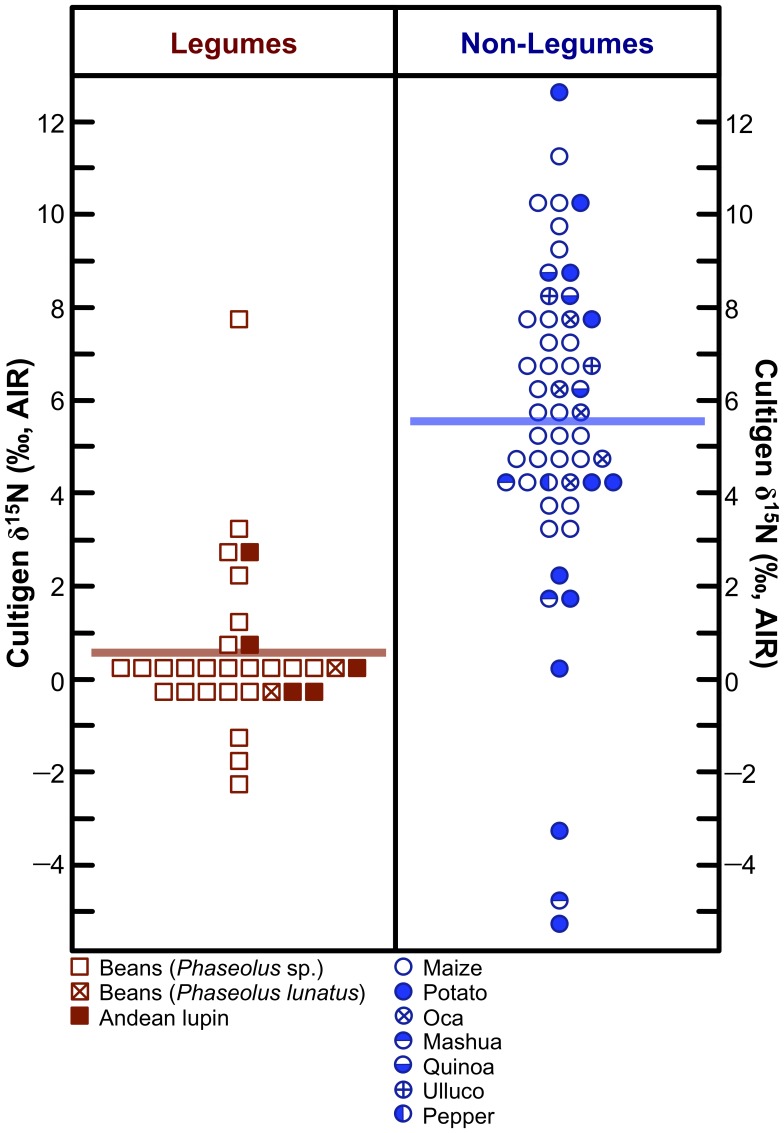
Dot-matrix plot of nitrogen isotopic compositions of legumes and non-legumes. Horizontal bars represent means. Increment = 0.5 ‰.

When maize is excluded, there were no significant differences in δ^13^C among cultigens (*F* [_7_], [_49_] = 0.3, *p* = 0.93), but there were for δ^15^N (maize included) (*F* [_8_], [_73_] = 9.7, *p*<0.001). Results of post-hoc Dunnett’s T3 test for δ^15^N differences among individual cultigen species are presented in Table 4. The three leguminous species were generally characterized by significantly lower δ^15^N values than non-leguminous species (Table 4); collectively, legumes were characterized by significantly lower δ^15^N values than non-legumes (Figure 6; *F* [_1]_, [_80_] = 51.8, *p*<0.001).

**Table 4:** Results of ANOVA post-hoc tests (Dunnett’s T3) for cultigen δ^15^N.

Cultigen %N	Bean (*P. lunatus*)	Andean lupin	Maize	Mashua	Oca	Potato	Quinoa	Ulluco
Bean (*Phaseolus* sp.)	0.860	1.000	**<0.001**	1.000	**0.003**	0.798	**0.028**	0.880
Bean (P. lunatus)	−	0.971	**<0.001**	1.000	**0.005**	0.479	**0.037**	0.121
Andean lupin	−	−	**<0.001**	1.000	**0.006**	0.802	**0.020**	0.060
Maize	−	−	−	0.696	1.000	0.983	0.855	0.917
Mashua	−	−	−	−	0.788	1.000	0.626	0.723
Oca	−	−	−	−	−	1.000	0.626	0.723
Potato	−	−	−	−	−	−	0.688	0.780
Quinoa	−	−	−	−	−	−	−	1.000

Values in boldface are statistically significant (*p*<0.05).

**Figure 7:**
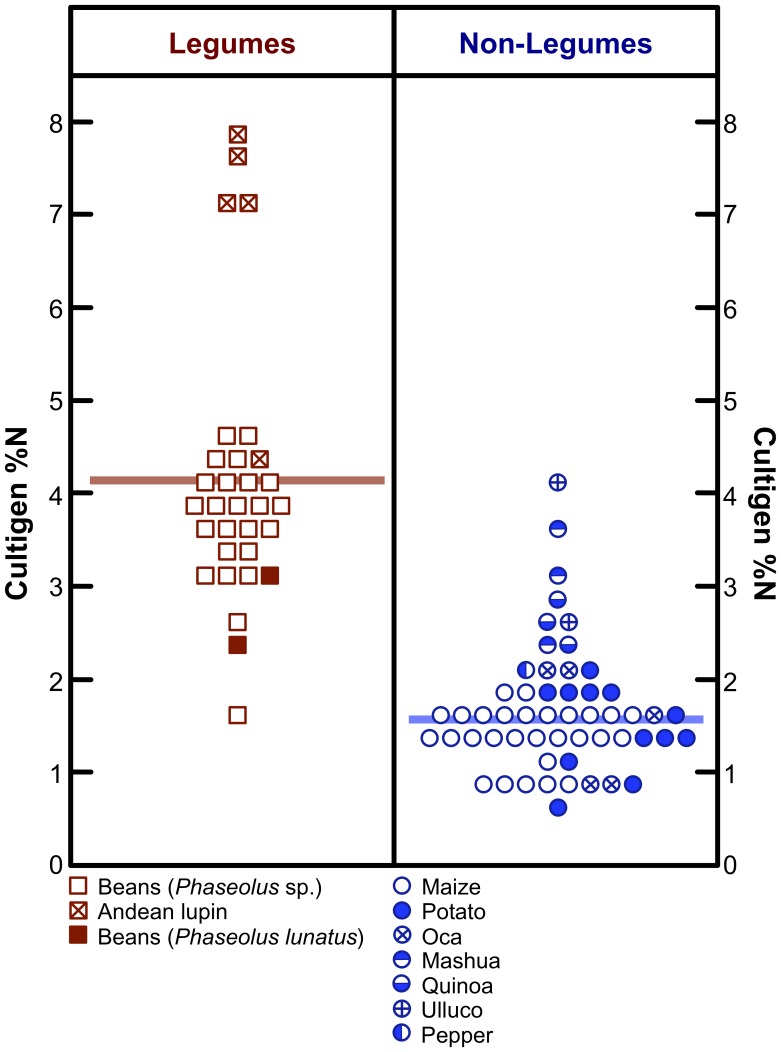
Dot-matrix plot of nitrogen content of legumes and non-legumes. Horizontal bars represent means. Increment = 0.25%.

Cultigen N content is presented in Table 3 and Figure 7. Mean %N for cultigens ranged from 1.2±0.2% (maize) to 6.8±1.3% (Andean lupin). Results of post-hoc Dunnett’s T3 test for differences between individual cultigen species in N content are presented in Table 5. The three leguminous species were characterized by significantly higher N contents than non-leguminous species (Table 5); collectively, legumes were characterized by significantly higher %N values than non-legumes (Figure 7; *F* [_1]_, [_80_] = 116.0, *p*<0.001).

**Table 5:** Results of ANOVA post-hoc tests (Dunnett’s T3) for cultigen N content.

Cultigen %N	Bean (*P. lunatus*)	Andean lupin	Maize	Mashua	Oca	Potato	Quinoa	Ulluco
Bean (*Phaseolus* sp.)	0.637	0.072	**<0.001**	0.869	**0.009**	**<0.001**	0.123	1.000
Bean (P. lunatus)	−	**0.037**	0.462	1.000	0.619	0.505	1.000	0.995
Andean lupin	−	−	**0.009**	**0.034**	**0.005**	**0.008**	**0.021**	0.295
Maize	−	−	−	0.232	0.981	0.992	0.101	0.566
Mashua	−	−	−	−	**0.019**	1.000	0.216	**0.009**
Oca	−	−	−	−	−	1.000	**0.033**	**0.001**
Potato	−	−	−	−	−	−	**0.033**	**0.001**
Quinoa	−	−	−	−	−	−	−	0.885

Values in boldface are statistically significant (*p*<0.05).

### Wild Plants

A total of 139 species were sampled primarily from ten sites distributed along an altitudinal transect from 10 to 4,070 masl. The number of taxa sampled and environmental variables for each of the sampling locations are presented in Table 6. The number of C_4_ plant taxa was generally higher at lower altitude sites receiving low amounts of rainfall. This fits with what is known about the global distribution of C_4_ plants [185].

**Table 6:** Environmental data for wild plant sampling sites and summary of number of C_3_ and C_4_ plant species sampled.

Site ID	Site Name	Latitude	Longitude	Altitude (masl)	MAP (mm)^1^	C_3_ Plant Taxa Sampled	C_4_ Plant Taxa Sampled
W1	Las Delicias	−8.1956	−78.9996	10	7	7	2
W2	Río Moche	−8.1267	−78.9963	33	5	9	1
W3	Ciudad Universitaria	−8.1137	−79.0373	38	6	2	0
W4	Cerro Campana	−7.9900	−79.0768	164	11	4	1
W5	La Carbonera	−8.0791	−78.8681	192	56	5	3
W6	Poroto	−8.0137	−78.7972	447	113	17	6
W7	Salpo 5	−8.0089	−78.6962	1181	143	0	2
W8	Salpo 4	−8.0047	−78.6726	1557	140	9	0
W9	Salpo 3	−8.0132	−78.6355	2150	141	16	0
W10	Salpo 2	−7.9973	−78.6481	2421	142	8	0
W11	Salpo 1	−8.0132	−78.6355	2947	171	9	1
W12	Stgo de Chuco	−8.1361	−78.1685	3041	702	21	1
W13	Cahuide	−8.2235	−78.3013	4070	591	15	0

1 Mean annual precipitation (MAP) estimated as described in the text.

The carbon and nitrogen isotopic compositions were measured for all 139 species. Foliar tissue was analyzed from all species, and additional tissues analyzed included: 112 stems, 28 roots, 51 flowers, and 62 seeds. Carbon and nitrogen isotopic compositions for wild plants are presented in Table 7 according to plant part. Foliar δ^13^C values for C_3_ plants ranged from −31.9 to −22.5 ‰, with a mean value of −27.6±1.9 ‰ (*n* = 122). Foliar δ^13^C values for C_4_ plants ranged from −15.6 to −11.6 ‰, with a mean value of −13.5±1.0 ‰ (*n* = 17). Foliar δ^15^N values for C_3_ plants ranged from −4.1 to 13.0‰, with a mean value of 3.7±4.0 ‰. Foliar δ^15^N values for C_4_ plants ranged from −3.2 to 15.0 ‰, with a mean value of 5.5±5.7 ‰. The single lichen analyzed (*Usnea andina*) was characterized by a δ^13^C value intermediate between C_3_ and C_4_ plants (−20.5 ‰) and a very low δ^15^N value (−6.5 ‰), consistent with previously reported results for lichens [71]–[73].

**Table 7:** Carbon and nitrogen isotopic compositions for all wild plant taxa sampled.

				Leaf		Stem		Root		Flowers		Seeds	
Taxonomic Name	Site ID	Altitude	Type	δ^13^C (‰)	δ^15^N (‰)	δ^13^C (‰)	δ^15^N (‰)	δ^13^C (‰)	δ^15^N (‰)	δ^13^C (‰)	δ^15^N (‰)	δ^13^C (‰)	δ^15^N (‰)
Eriochloa mutica	W1	10	Grass	−11.6	−1.5	−11.7	1.6	−	−	−	−	−12.1	−0.2
Distichia spicata	W1	10	Grass	−14.9	−3.2	−	−	−	−	−	−	−	−
Baccharis glutinosa	W1	10	Shrub	−27.4	3.3	−26.6	5.1	−	−	−27.0	4.1	−26.9	4.2
Rauvolfia sp.	W1	10	Shrub	−28.0	9.8	−27.9	11.0	−	−	−	−	−	−
Plantago major^1^	W1	10	Herb	−28.6	7.5	−27.5	7.9	−26.7	8.6	−	−	−	−
Typla angustifolia	W1	10	Herb	−29.3	1.3	−	−	−28.7	2.6	−	−	−	−
Blumea crispata^1^	W1	10	Herb	−29.8	13.7	−30.4	13.7	−30.5	11.6	−	−	−	−
Rosippa nastrutium aquaticum^1^	W1	10	Herb	−30.1	12.5	−	−	−30.0	11.4	−	−	−	−
Oxalis corniculata	W1	10	Herb	−30.6	7.1	−31.0	6.0	−31.2	4.7	−	−	−	−
Paspalum racemosum	W2	33	Grass	−12.7	0.8	−12.8	11.7	−	−	−	−	−	−
Salix humboldtiana	W2	33	Tree	−26.4	5.2	−26.5	4.4	−	−	−	−	−	−
Phyla nodiflora	W2	33	Herb	−27.7	6.5	−26.8	5.1	−27.1	2.8	−26.7	7.9		
Melochia lupulina	W2	33	Shrub	−28.3	6.9	−27.6	6.4	−	−	−28.6	6.8	−	−
Ipomoea alba	W2	33	Herb	−28.7	9.3	−28.1	8.1	−	−	−	−	−27.3	8.9
Persea americana	W2	33	Tree	−28.8	1.9	−26.8	7.0	−	−	−	−	−	−
Ambrosia peruviana	W2	33	Herb	−29.6	2.2	−30.0	1.7	−	−	−	−	−	−
Arundo donax^1^	W2	33	Grass	−30.3	8.5	−30.1	10.2	−	−	−	−	−	−
Acacia huarango^2^	W2	33	Shrub	−31.0	3.5	−30.0	2.3	−	−	−29.8	3.4	−	−
Psittacanthus obovatus	W2	33	Shrub (Parasitic)	−31.9	5.1	−30.6	6.2	−	−	−	−	−	−
Prosopis pallida^2^	W3	38	Tree	−27.9	4.0	−28.9	1.5	−	−	−29.1	5.8	−	−
Acacia macracantha^2^	W3	38	Tree	−30.7	8.3	−30.1	6.8	−	−	−30.5	8.6	−28.9	5.1
Tillandsia usneoides	W4	164	Epiphyte	−13.6	3.7	−14.2	1.9	−13.9	14.5	−13.6	0.0	−	−
Cryptocarpus pyriformis	W4	164	Shrub	−22.5	10.3	−22.2	10.4	−	−	−	−	−22.4	12.1
Trixis cacalioides	W4	164	Shrub	−26.6	9.2	−26.0	7.6	−	−	−	−	−25.7	9.4
Scutia spicata	W4	164	Shrub	−27.1	4.9	−25.7	4.4	−	−	−	−	−	−
Capparis angulata	W4	164	Shrub	−27.3	10.0	−27.7	10.7	−	−	−	−	−26.0	11.6
Paspalidium paladivagum	W5	192	Grass	−12.5	10.5	−12.8	11.0	−12.0	11.7	−	−	−12.3	13.4
Amaranthus celosiodes	W5	192	Herb	−13.1	9.1	−12.5	11.0	−	−	−13.5	10.9	−12.2	8.6
Tribulus terrestris	W5	192	Herb	−15.6	11.8	−16.2	14.4	−	−	−	−	−14.0	13.6
Hydrocotyle bonariensis	W5	192	Herb	−26.5	9.0	−	−	−	−	−	−	−	−
Cestrum auriculatum	W5	192	Shrub	−26.9	10.6	−26.8	8.5	−	−	−	−	−27.0	12.1
Cucumis dipsaceus	W5	192	Herb	−27.4	5.6	−26.8	4.2	−	−	−27.0	5.5	−28.1	6.5
Argemone subfusiformis	W5	192	Herb	−28.8	6.9	−28.1	6.3	−28.8	6.2	−28.9	5.7	−	−
Picrosia longifolia	W5	192	Herb	−30.6	5.3	−30.5	1.1	−	−	−30.0	9.3	−	−
Cyperus corymbosus	W6	447	Sedge	−13.1	8.3	−11.2	8.8	−11.7	7.8	−14.2	9.1	−	−
Echinochloa crusgalli^1^	W6	447	Grass	−13.4	2.8	−13.8	2.8	−	−	−	−	−13.7	3.9
Cynodon dactylon^1^	W6	447	Grass	−13.9	0.8	−	−	−	−	−14.1	1.2	−	−
Sorghum halepense^1^	W6	447	Grass	−14.0	2.5	−15.0	4.7	−	−	−	−	−13.1	3.7
Trianthema portulacastrum	W6	447	Herb	−14.2	17.3	−13.7	12.3	−	−	−	−	−	−
Amaranthus spinosus	W6	447	Herb	−14.4	13.3	−13.8	16.1	−	−	−14.0	15.3	−14.4	15.3
Gynerium sagittatum	W6	447	Grass	−25.8	2.7	−25.1	2.3			−25.6	5.3		
Alternanthera halimifolia	W6	447	Herb	−26.0	8.4	−26.1	9.2	−	−	−26.1	8.2	−	−
Cissus sicyoides	W6	447	Vine	−26.6	10.9	−26.1	12.4	−	−	−	−	−25.1	11.9
Dalea onobrychis^2^	W6	447	Herb	−27.2	8.7	−27.4	7.8	−	−	−	−	−26.8	7.4
Cleome spinosa	W6	447	Herb	−27.3	9.0	−27.1	9.8	−	−	−	−	−26.9	9.8
Crotalaria incae^2^	W6	447	Shrub	−27.3	0.2	−26.6	−2.4	−	−	−25.4	1.0	−26.0	−0.8
Ludwigia octovalvis^2^	W6	447	Herb	−27.5	0.6	−26.9	1.3	−	−	−	−	−	−
Passiflora foetida	W6	447	Vine	−27.5	9.5	−27.2	1.7	−	−	−27.5	7.8	−	−
Wedelia latilofolia	W6	447	Shrub	−28.0	6.4	−27.3	4.8	−	−	−26.4	8.1	−	−
Baccharis salicifolia	W6	447	Shrub	−28.3	6.5	−27.2	8.4	−	−	−	−	−27.1	8.0
Waltheria ovata	W6	447	Shrub	−28.4	6.1	−28.2	5.9	−	−	−27.7	6.0	−	−
Verbena littoralis	W6	447	Herb	−28.8	7.9	−28.4	5.8	−	−	−	−	−27.7	7.3
Cyperus odoratus	W6	447	Sedge	−28.8	9.2	−27.6	10.1	−	−	−28.0	10.2	−	−
Mimosa pigra	W6	447	Shrub	−29.3	1.7	−28.5	0.3	−	−	−	−	−29.1	1.3
Cajanus cajan^1, 2^	W6	447	Tree	−29.6	−1.4	−28.4	−	−	−	−28.3	0.3	−27.6	−0.7
Polygonum hydropiperoides	W6	447	Herb	−30.2	6.8	−30.6	6.7	−	−	−	−	−27.2	8.1
Mimosa albida^2^	W6	447	Shrub	−30.5	−0.8	−30.1	−1.5	−	−	−	−	−28.8	1.2
Melinis repens^1^	W7	1181	Grass	−13.3	5.6	−13.4	7.3	−	−	−	−	−14.5	3.1
Cenchrus myosuroides	W7	1181	Grass	−13.3	5.7	−	−	−	−	−	−	−	−
Dicliptera peruviana	W8	1557	Herb	−24.7	3.9	−26.3	3.2	−	−	−	−	−25.0	3.2
Tournefortia microcalyx	W8	1557	Shrub	−26.0	6.4	−26.2	6.0	−	−	−25.6	7.2	−	−
Ophryosporus peruvianus	W8	1557	Shrub	−26.7	2.9	−23.9	1.8	−	−	−	−	−24.0	2.6
Alternanthera porrigens	W8	1557	Herb	−27.8	2.8	−27.2	2.2	−	−	−	−	−25.7	6.7
Asclepias curassavica	W8	1557	Shrub	−28.9	2.6	−28.7	4.2	−	−	−28.8	0.4	−28.0	0.2
Boerhavia erecta	W8	1557	Herb	−29.3	9.1	−28.3	9.2	−	−	−	−	−	−
Centaurea melitensis	W8	1557	Herb	−29.5	0.5	−29.8	0.2	−	−	−	−	−28.9	1.7
Mentzelia aspera	W8	1557	Herb	−30.0	1.0	−27.6	6.8	−	−	−29.5	1.3	−	−
Sida spinosa	W8	1557	Herb	−30.1	3.1	−30.1	4.7	−	−	−31.3	1.7	−	−
Rubus robustus	W9	2150	Shrub	−25.0	3.0	−24.4	2.7	−	−	−	−	−	−
Puya sp.	W9	2150	Succulent	−25.4	−0.7	−	−	−	−	−	−	−	−
Barnadesia dombeyana	W9	2150	Shrub	−26.0	−2.0	−25.4	−0.3	−	−	−25.7	−2.7	−	−
Iochroma edule	W9	2150	Shrub	−26.1	8.5	−25.8	7.6	−	−	−	−	−25.4	7.3
Eupatorium sp.	W9	2150	Herb	−26.8	2.5	−	−	−	−	−	−	−	−
Capparis scabrida	W9	2150	Shrub	−26.8	1.3	−26.3	0.9	−	−	−26.5	2.2	−	−
Vasquezia oppositifolia	W9	2150	Herb	−27.0	−1.6	−	−	−	−	−	−	−26.9	−1.3
Stipa ichu	W9	2150	Grass	−27.0	0.3	−	−	−27.4	0.2	−27.3	0.6	−	−
Lupinus sp.^2^	W9	2150	Herb	−27.1	1.4	−27.1	3.4	−	−	−26.6	3.2	−26.7	0.8
Alonsoa meridionalis	W9	2150	Herb	−27.5	1.3	−26.4	−1.9	−	−	−	−	−25.9	0.1
Bromus catharticus	W9	2150	Grass	−27.8	1.1	−29.3	−1.3	−	−	−	−	−27.5	−0.7
Baccharis sp.	W9	2150	Shrub	−28.9	−1.1	−28.8	0.1	−	−	−29.5	0.5	−	−
Minthostachys mollis	W9	2150	Herb	−29.0	0.5	−28.1	−1.6	−	−	−27.2	0.1	−	−
Satureja sp.	W9	2150	Herb	−30.2	−3.2	−	−	−	−	−29.8	−2.3	−	−
Achyrocline alata	W9	2150	Shrub	−30.3	0.3	−27.8	1.2	−	−	−27.5	2.0	−	−
Polypogon sp.	W9	2150	Grass	−31.1	−5.3	−27.9	−4.4	−31.0	2.4	−27.8	−3.7	−	−
Browallia americana	W10	2421	Herb	−25.4	−1.6	−26.8	−2.5	−	−	−25.7	−0.8	−	−
Coniza sp.	W10	2421	Herb	−26.7	6.1	−26.1	4.0	−	−	−	−	−	−
Heliotropium sp.	W10	2421	Herb	−26.9	3.7	−28.4	2.2	−	−	−	−	−28.2	3.2
Caesalpina spinosa^2^	W10	2421	Tree	−27.4	2.7	−27.7	−0.4	−	−	−	−	−25.1	0.0
Oenothera rosea	W10	2421	Herb	−27.4	4.9	−27.9	4.6	−	−	−	−	−27.4	2.9
Avena sterilis^1^	W10	2421	Grass	−27.5	2.3	−27.2	2.1	−27.0	0.0	−	−	−22.5	2.2
Berberis sp.	W10	2421	Shrub	−27.7	1.1	−24.6	1.9	−	−	−	−	−26.7	1.9
Alternanthera sp.	W10	2421	Herb	−28.3	−2.9	−27.5	−3.0	−	−	−	−	−27.2	−0.8
Pennisetum purpurem^1^	W11	2947	Grass	−12.5	7.2	−12.8	6.6	−	−	−	−	−15.5	7.1
Ruellia floribunda	W11	2947	Herb	−23.7	4.5	−24.0	1.9	−	−	−23.5	4.9	−	−
Schinus molle	W11	2947	Tree	−24.6	2.3	−23.4	0.3	−	−	−21.3	0.8	−	−
Spartium junceum^1, 2^	W11	2947	Shrub	−26.5	1.1	−27.1	−1.1	−	−	−23.7	−1.3	−25.4	0.8
Acacia aroma^2^	W11	2947	Tree	−26.8	9.6	−26.6	9.6	−	**−**	−26.6	10.1	−	**−**
Croton ovalifolius	W11	2947	Shrub	−27.0	7.4	−27.6	5.8	−	−	−	−	−	−
Leonotis nepetifolia^1^	W11	2947	Shrub	−28.0	2.2	−	−	−	−	−27.2	3.0	−26.1	2.0
Lycianthes lycioides	W11	2947	Shrub	−28.0	−0.3	−	−	−	−	−	−	−24.3	2.0
Phenax hirtus	W11	2947	Shrub	−28.3	2.5	−29.1	7.1	−	−	−	−	−28.5	6.9
Inga feulleu^2^	W11	2947	Tree	−28.9	0.3	−27.6	−0.8	−	−	−	−	−27.1	1.1
Andropogon sp.	W12	3041	Grass	−13.5	−1.6	−	−	−13.2	−1.0	−	−	−	−
Sebastiania obtusifolia	W12	3041	Shrub	−23.7	0.8	−24.7	0.0	−	−	−	−	−24.0	2.5
Lupinus aridulus^2^	W12	3041	Herb	−24.3	2.0	−23.7	2.2	−	−	−22.7	4.0	−22.0	5.4
Silybum marianum^1^	W12	3041	Herb	−25.9	2.2	−25.8	1.6	−	−	−	−	−25.1	2.2
Phrygilanthus sp.	W12	3041	Shrub (Parasitic)	−25.9	−0.5	−24.7	7.3	−	−	−	−	−	−
Solanum amotapense	W12	3041	Shrub	−25.9	7.9	−25.2	5.1	−	−	−	−	−24.7	8.3
Acacia sp.^2^	W12	3041	Tree	−26.2	−1.0	−25.0	−2.5	−	−	−	−	−	−
Baccharis serpifolia	W12	3041	Shrub	−26.4	2.2	−27.1	1.5	−	−	−26.9	1.0	−	−
Aristida adsensionis	W12	3041	Grass	−26.5	−2.6	−26.2	−2.0	−	−	−26.7	−1.0	−	−
Baccharis emarginata	W12	3041	Shrub	−26.5	−0.2	−25.4	0.1	−	−	−	−	−	−
Brassica campestris	W12	3041	Herb	−27.1	2.3	−	−	−	−	−	−	−25.4	4.3
Mauria sp.	W12	3041	Tree	−27.2	6.1	−25.8	3.1	−	−	−	−	−	−
Solanum agrimoniaefolium	W12	3041	Shrub	−28.0	6.0	−28.2	3.8	−	−	−	−	−26.6	4.1
Salvia punctata	W12	3041	Herb	−28.1	−3.5	−	−	−	−	−27.7	−2.1	−27.3	−1.7
Duranta sp.	W12	3041	Shrub	−28.4	1.3	−27.6	0.8	−	−	−	−	−	−
Flourensia cajabambensis	W12	3041	Shrub	−28.6	2.9	−27.5	2.7	−	−	−	−	−29.4	2.5
Marrubium vulgare	W12	3041	Herb	−28.8	3.8	−26.6	1.4	−27.9	1.0	−26.9	4.0	−	−
Scutellaria sp.	W12	3041	Herb	−28.8	2.2	−28.6	0.6	−	−	−	−	−	−
Viguiera peruviana	W12	3041	Shrub	−28.9	5.3	−27.3	4.6	−	−	−	−	−26.6	5.4
Jungia rugosa	W12	3041	Shrub	−28.9	1.4	−26.8	1.1	−	−	−27.1	2.7	−	−
Saccellium sp.	W12	3041	Shrub	−29.0	2.0	−27.3	1.0	−	−	−	−	−	−
Baccharis libertadensis	W12	3041	Shrub	−29.6	3.8	−28.4	1.9	−	−	−	−	−	−
Usnea andina	W13	4070	Lichen	−20.5	−6.5	−	−	−	−	−	−	−	−
Astragalus garbancillo^2^	W13	4070	Shrub	−24.6	4.2	−25.3	3.0	−25.1	3.8	−23.8	3.9	−22.5	5.4
Luzula sp.	W13	4070	Sedge	−25.1	0.9	−	−	−25.0	3.9	−25.1	3.2	−	−
Distichia muscoides	W13	4070	Grass	−25.3	4.4	−	−	−25.2	2.9	−	−	−	−
Muehlenbeckia sp.	W13	4070	Herb	−25.3	6.3	−	−	−25.7	4.9	−	−	−	−
Urtica sp.	W13	4070	Shrub	−25.5	11.9	−25.1	9.0	−26.0	9.4	−	−	−26.6	11.9
Agrostis breviculmis	W13	4070	Grass	−25.9	2.1	−	−	−26.0	4.1	−25.5	2.4	−	−
Chuquiraga spinosa	W13	4070	Shrub	−26.0	−0.5	−24.9	−1.1	−24.4	−1.4	−24.3	−0.7	−24.4	−0.2
Werneria nubigena	W13	4070	Herb	−26.2	1.3	−	−	−25.8	1.8	−	−	−	−
Festuca dolichopylla	W13	4070	Grass	−26.3	−1.8	−	−	−25.4	−0.3	−	−	−26.5	3.6
Hypochaeris sp.	W13	4070	Herb	−26.6	7.3	−	−	−26.9	8.2	−	−	−	−
Plantago tubulosa	W13	4070	Herb	−26.9	−5.2	−	−	−26.0	−3.0	−	−	−	−
Stipa mucronata	W13	4070	Grass	−27.7	−1.4	−	−	−26.2	1.5	−	−	−26.5	1.5
Stenandrium dulce	W13	4070	Herb	−28.4	0.6	−	−	−	−	−	−	−	−
Senecio nutans	W13	4070	Shrub	−29.4	6.2	−28.6	5.0	−27.6	5.3	−	−	−	−

1. Species is invasive or introduced.

2. Member of the family Fabaceae (legume).

There were no significant differences in foliar δ^15^N among plant functional groups (*F* [_3]_, [_132_] = 1.8, *p* = 0.15). Foliar δ^13^C differed significantly among plant functional groups (*F* [_5]_, [_130_] = 195.0, *p*<0.001), although this was driven by differences between C_3_ and C_4_ groups; there were no significant differences in foliar δ^13^C between plant functional groups within C_3_ and C_4_ groups (Table 8).

**Table 8:** Results of ANOVA post-hoc tests (Dunnett’s T3) for foliar δ^13^C between plant functional groups.

Foliar δ^13^C	C_3_ Grass/Sedge	C_4_ Herb/Shrub	C_3_ Herb/Shrub	Tree	Vine
C_4_ Grass/Sedge	**<0.001**	0.999	**<0.001**	**<0.001**	**<0.001**
C_3_ Grass/Sedge	−	**<0.001**	0.993	1.000	1.000
C_4_ Herb/Shrub	−	−	0.999	**<0.001**	**<0.001**
C_3_ Herb/Shrub	−	−	−	0.997	0.994
Tree	−	−	−	−	1.000

Values in boldface are statistically significant (*p*<0.05).

**Figure 8:**
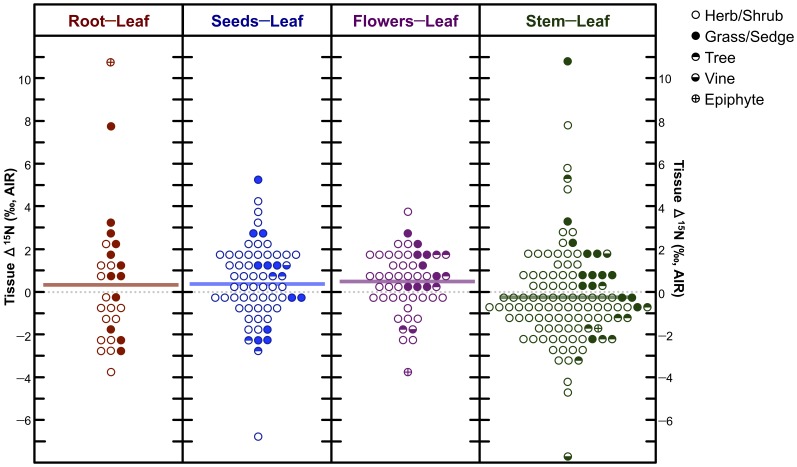
Dot-matrix plot of differences in nitrogen isotopic composition between foliar and other tissues (Δ^15^N). Horizontal bars represent means. Increment = 0.5 ‰.

**Figure 9:**
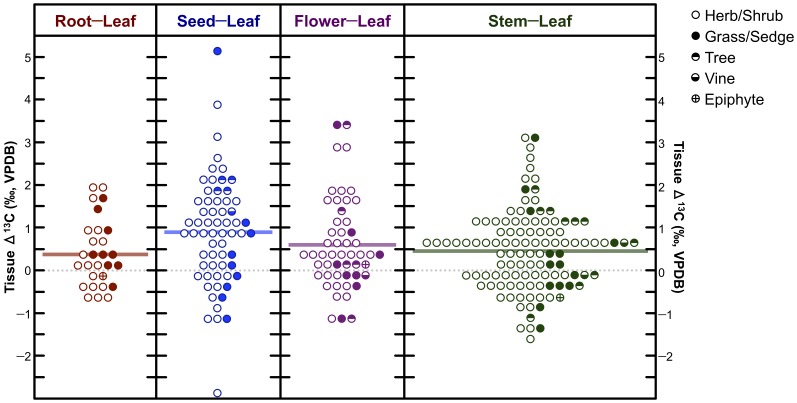
Dot-matrix plot of differences in carbon isotopic composition between foliar and other tissues (Δ^13^C). Horizontal bars represent means. Increment = 0.5 ‰.

There was no clear pattern of intraplant variation in δ^15^N (Figure 8) with differences in δ^15^N between tissues (Δ^15^N) being highly variable: Δ^15^N_stem−leaf_ = −0.3±2.3 ‰, Δ^15^N_root−leaf_ = 0.4±3.1 ‰, Δ^15^N_flower−leaf_ = 0.5±1.4 ‰, Δ^15^N_seed−leaf_ = 0.5±1.7 ‰. Conversely, foliar tissue was typically characterized by lower δ^13^C values than all other tissues analyzed (Figure 9), and intraplant variation was generally smaller: Δ^13^C_stem−leaf_ = 0.5±0.9 ‰, Δ^13^C_root−leaf_ = 0.4±0.8 ‰, Δ^13^C_flower−leaf_ = 0.6±1.0 ‰, Δ^13^C_seed−leaf_ = 0.5±1.7 ‰. For C_4_ plants (*n* = 17), there was no clear pattern of intraplant variation in δ^13^C: Δ^13^C_stem−leaf_ = 0.0±0.8 ‰, Δ^13^C_root−leaf_ = 0.5±0.7 ‰, Δ^13^C_flower−leaf_ = −0.3±0.6 ‰, Δ^13^C_seed−leaf_ = −0.2±1.3 ‰.

Foliar nitrogen isotopic compositions for wild legumes (Fabaceae) were highly variable, ranging from −1.4 to 9.6 ‰. Among *Acacia* trees and shrubs alone, foliar δ^15^N values ranged from −1.0 to 9.6 ‰, suggesting that some species are not engaged in active N_2_-fixation. While wild legumes were characterized by lower foliar δ^15^N values relative to non-legumes (4.1±4.4 ‰, *n* = 119 for non-legumes; 2.7±3.4 ‰, *n* = 17 for legumes), this difference was not statistically significant (*F* [_1]_, [_134_] = 1.8, *p* = 0.18).

**Figure 10:**
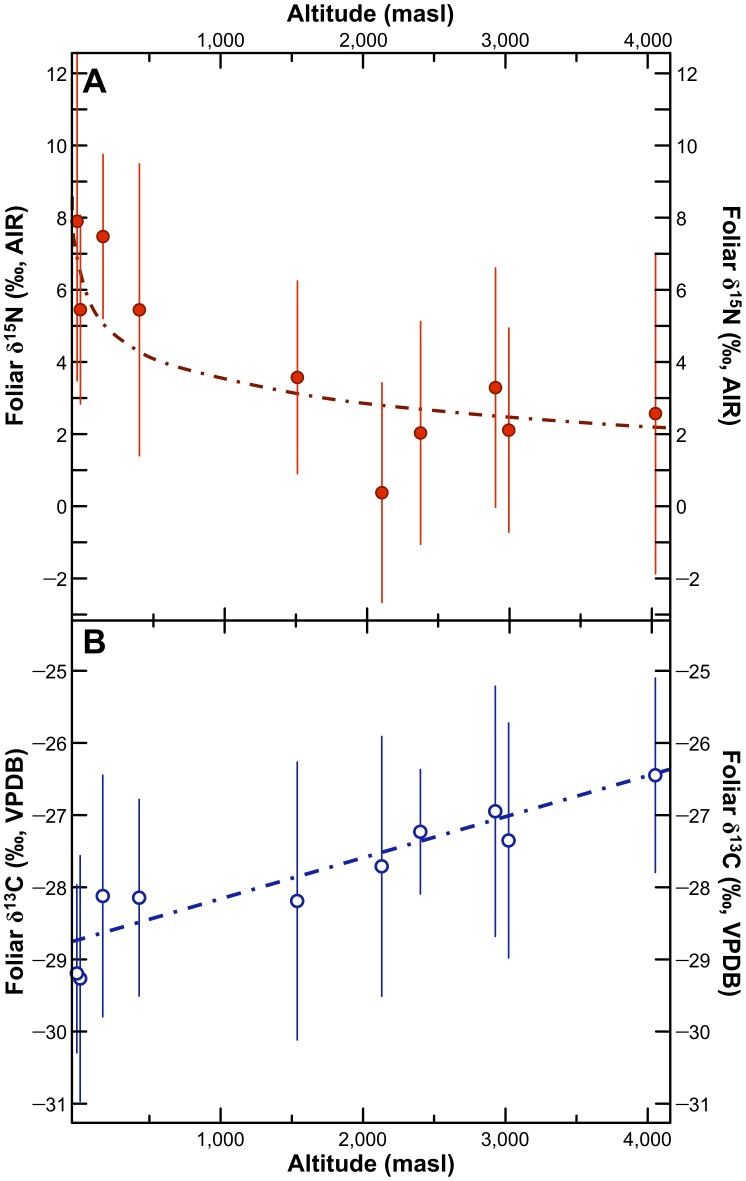
Bivariate plots of foliar δ^15^N and altitude (A) and foliar δ^13^C (B) for C_3_ plants only. Points represent means ±1σ for sites with ≥5 C_3_ plant species sampled. Equation for δ^15^N and altitude: *y* = 10.3– log*x*, *r* ^2^ = 0.71; *p* = 0.002. Equation for δ^13^C and altitude: *y* = *x*/1,733–28.8, *r* ^2^ = 0.85; *p*<0.001.

**Figure 11:**
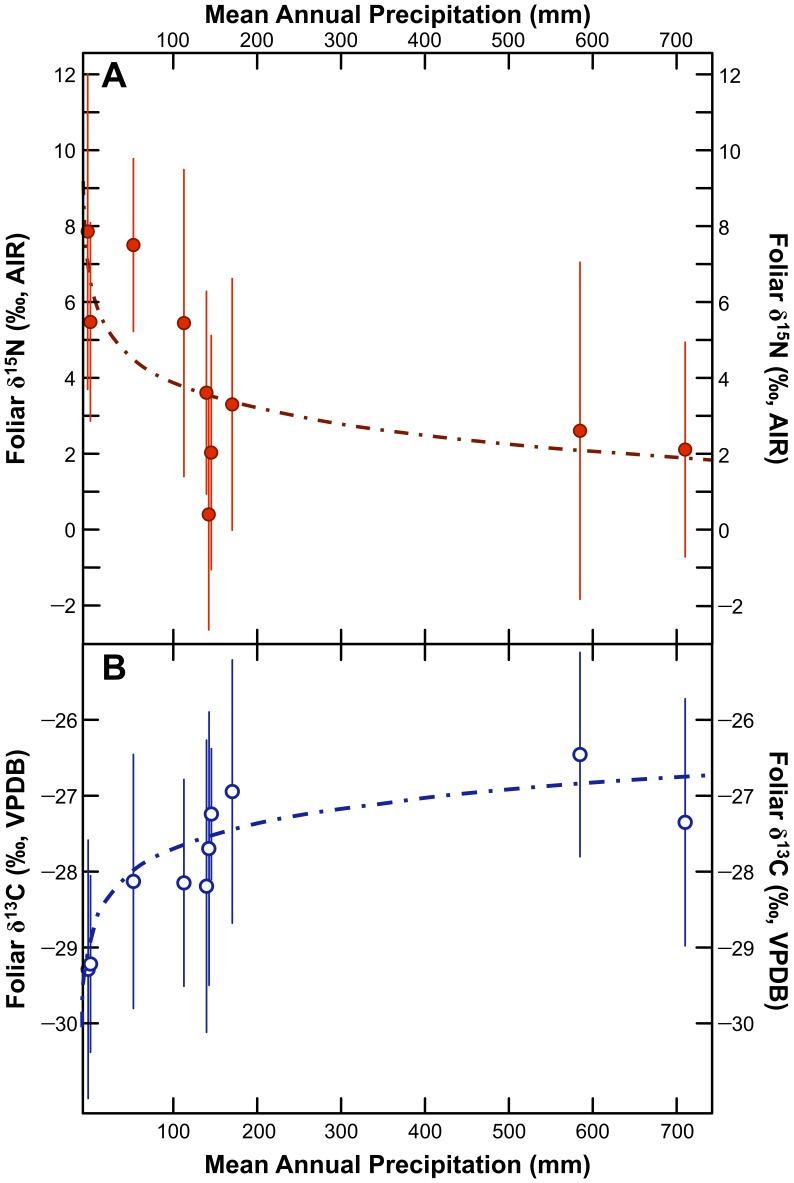
Bivariate plots of foliar δ^15^N and mean annual precipitation (A) and foliar δ^13^C (B) for C_3_ plants only. Points represent means ±1σ for sites with ≥5 C_3_ plant species sampled. Equation for δ^15^N and MAP: *y* = 8.8–1.1 log*x*, *r* ^2^ = 0.49; *p* = 0.03. Equation for δ^13^C and MAP: *y* = −30.1+0.5 log*x*, *r* ^2^ = 0.81; *p*<0.001.

**Figure 12:**
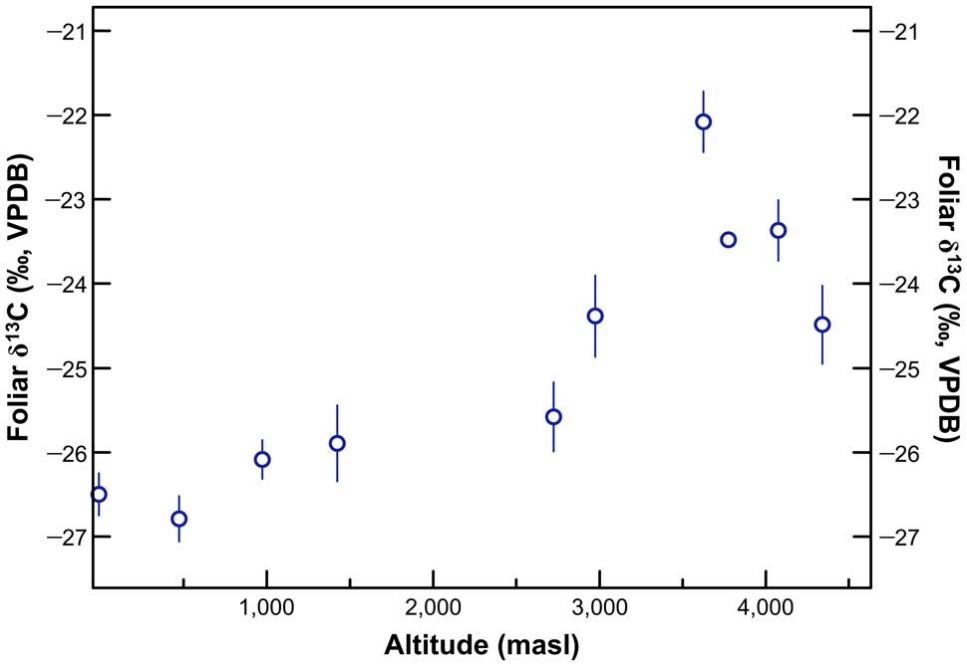
Bivariate plot of altitude and foliar δ^13^C for plants collected in northern Chile [**14**].

Mean wild C_3_ plant foliar δ^13^C and δ^15^N values for sampling locations with ≥5 species sampled are presented in Table 9. Mean foliar carbon and nitrogen isotopic compositions for these sites are plotted against altitude in Figure 10 and estimated mean annual precipitation in Figure 11. Mean foliar δ^15^N values at low altitude sites were 2 to 8 ‰ higher than mean foliar δ^15^N values at high altitude sites. Foliar δ^15^N was negatively correlated with mean annual precipitation (Spearman’s *ρ* = −0.770, *p* = 0.009) and altitude (Spearman’s *ρ* = −0.782, *p* = 0.008). Foliar δ^13^C was positively correlated with mean annual precipitation (Spearman’s *ρ* = 0.879, *p* = 0.001) and altitude (Spearman’s *ρ* = 0.903, *p*<0.001). For comparative purposes, mean plant δ^13^C values for sites sampled along an altitudinal transect in northern Chile are presented in Figure 12 [14].

**Table 9:** Mean (±1σ) isotopic and elemental compositions for sampling locations with >3 plant species sampled (data for C_3_ plants only).

Site ID	Latitude	Longitude	Altitude (masl)	MAP (mm)^1^	*n* ^2^	δ^13^C (‰, VPDB)	δ^15^N (‰, AIR)
W1	−8.1956	−78.9996	10	7	7	−29.1±1.2	7.9±4.5
W2	−8.1267	−78.9963	33	5	9	−29.2±1.7	5.5±2.6
W5	−8.0791	−78.8681	192	56	5	−28.1±1.7	7.5±2.2
W6	−8.0137	−78.7972	447	113	17	−28.1±1.4	5.4±4.0
W8	−8.0047	−78.6726	1557	140	9	−28.1±1.9	3.6±2.7
W9	−8.0132	−78.6355	2150	141	16	−27.6±1.8	0.4±3.0
W10	−7.9973	−78.6481	2421	142	8	−27.2±0.8	2.0±3.1
W11	−8.0132	−78.6355	2947	171	9	−26.9±1.8	3.3±3.3
W12	−8.1361	−78.1685	3041	702	21	−27.3±1.6	2.1±2.8
W13	−8.2235	−78.3013	4070	591	15	−26.0±2.0	2.0±4.9

1. Mean annual precipitation (MAP) estimated as described in the text.

2. Number of C_3_ plant species sampled.

### Marine Plants

The carbon and nitrogen isotopic compositions were determined for a total of 25 marine plant samples from five species. Mean δ^13^C and δ^15^N values for marine plants are presented in Table 10. Mean δ^13^C values for marine plants ranged from −18.7±0.7 ‰ (*Gymnogongrus furcellatus*) to −14.2±1.2 ‰ (*Grateloupia doryphora*). Mean δ^15^N values for marine plants ranged from 2.5±0.9 ‰ (*Gymnogongrus furcellatus*) to 7.8±0.1 ‰ (*Cryptopleura cryptoneuron*). Overall, marine plants were characterized by δ^13^C values that were intermediate between C_3_ and C_4_ plant isotopic compositions, although more similar to the latter. In comparison to wild plants growing at the three sites located closest to the coast, marine plants were not characterized by significantly higher δ^15^N values when the plants from the three terrestrial sites are treated separately (*F* [_3]_, [_39_] = 0.5, *p* = 0.71) or grouped together (*F* [_1]_, [_41_]<0.1, *p* = 0.91).

**Table 10:** Mean (±1σ) isotopic and elemental compositions for marine algae.

Taxonomic Name	Type	*n*	δ^13^C (‰, VPDB)	δ^15^N (‰, AIR)	%C	%N
*Ulva lactuca*	Chlorophyta	5	−14.3±0.4	6.4±0.1	29.2±0.4	3.6±0.2
*Gymnogongrus furcellatus*	Rhodophyta	5	−18.7±0.7	2.5±0.9	23.3±2.4	2.1±0.2
*Grateloupia doryphora*	Rhodophyta	5	−14.2±1.2	6.8±0.3	29.9±1.0	3.2±0.1
*Gigartina chamissoi*	Rhodophyta	5	−16.7±1.0	5.4±0.5	25.7±0.4	2.7±0.1
*Cryptopleura cryptoneuron*	Rhodophyta	5	−18.4±0.4	7.8±0.1	21.2±1.6	2.8±0.4

## Discussion

### Cultigens

The carbon isotopic composition of maize was ∼2 ‰ more enriched in ^13^C than wild C_4_ plants (all tissues), similar to previously determined values for other parts of the world [186], [187]. This suggests that a δ^13^C value of −10.3 ‰ (adjusted by +1.5 ‰ for the Suess Effect [188], [189]) would be appropriate for paleodietary models in the central Andes. There may, however, be some small-scale environmental effects on maize δ^13^C values along an altitudinal gradient as discussed in more detail below.

For the most part, the δ^15^N values of the modern cultigens presented in this study should be interpreted cautiously with respect to paleodietary studies. The primary factor influencing the nitrogen isotopic composition of plant tissues is the N source, and it cannot be assumed that modern N sources are directly analogous to those used in antiquity. The nitrogen isotopic composition of locally grown produce sold in Andean markets today may be influenced by chemical fertilizers (which cause plants to have relatively low nitrogen isotopic compositions) or by animal manures (e.g. sheep, cow, pig) that would not have been available in the region prior to the arrival of the Spanish. The same is true for nitrogen isotopic data obtained from modern agricultural plants globally, and as a general rule, the limitations of these data must be recognized. Nevertheless, some patterns are likely to be broadly applicable.

In contrast to the vast majority of published literature [27], [138]–[140], [190]–[200], Warinner et al. [187] showed very little distinction between the nitrogen isotopic composition of Mesoamerican legumes and non-legumes, suggesting that the assumption of lower δ^15^N values in legumes in that region is tenuous. Where the potential effects of nitrogenous fertilizers on legume δ^15^N values are unknown (as is the case for the data presented by Warriner et al. [187]), the interpretation of δ^15^N values in legumes and non-legumes is not straightforward. While there was some overlap in δ^15^N values between legumes and non-legumes in this study, leguminous cultigens had significantly higher N contents (Figure 7; Table 5) and significantly lower δ^15^N values (Figure 6; Table 4) than non-legumes.

Aside from the differences in δ^15^N between legumes and non-legumes, it is very difficult to generalize the δ^15^N values for cultigens in this study. Nitrogen isotopic compositions were highly variable, particularly for potato, which most likely reflected variable local growing conditions (soil fertility, type of manure used) rather than any biochemical or physiological process specific to any particular plant species. Ultimately, the best source of baseline isotopic data for paleodietary studies may be from archaeobotanical remains [27], [201]–[203], provided that preservation of original carbon and nitrogen isotopic compositions can be demonstrated. Considerable work has been done in this regard for the isotopic composition of bone collagen [204]–[208] and to a lesser extent hair keratin [209], but a solid set of parameters for detecting preservation versus alteration of original plant carbon and nitrogen isotopic compositions have not yet been determined. The excellent organic preservation at many archaeological sites on the coasts of Peru and Chile provides the potential for such analyses to be conducted on botanical remains.

### Wild Plants

#### Plant Functional Group

There were no clear distinctions between different plant functional groups (grass, herb, shrub, tree, vine) with respect to either carbon or nitrogen isotopic compositions. While some systematic variation may be expected due to variable nitrogen acquisition strategies (e.g. rooting depth) or differential distribution of biomolecules with distinct isotopic compositions, the diverse range of environmental conditions from which plants were sampled likely served to blur any isotopic distinctions between functional groups. Moreover, the sample sizes for different plant functional groups within any one site were too small for meaningful comparisons to be made.

There was no consistent pattern in plant δ^15^N with respect to leguminous trees and shrubs, with some species having foliar δ^15^N values close to 0 ‰, and others having relatively high δ^15^N values. Previous studies have similarly found conflicting patterns of relatively high and low δ^15^N values in leguminous trees. Codron et al. [13] found no clear distinction between leguminous and non-leguminous trees at a regional scale in South Africa. Aranibar et al. [178] did not observe significant amounts of N_2_-fixation among leguminous trees in an arid region of southern Africa, with trees growing at the most arid sites showing no evidence of N_2_-fixation. Fruit-bearing trees of the genus *Prosopsis* (often called huarango or algarrobo) are suggested to have been an important food source for various groups in the Andean region [64], [210]. Catenazzi and Donnelly [28] found δ^15^N values typical of N_2_-fixing trees (ca. 0 ‰) in *Prosopis pallida* from the Sechura Desert of northern Peru. Conversely, on the basis of the isotopic data recorded in this study for leguminous trees in the Moche River Valley, the assumption that *Prosopis* would be characterized by significantly lower δ^15^N values relative to other plants is tenuous. Given the potential importance of these foods in the diet, a more extensive study of the nitrogen isotopic composition of central Andean leguminous trees would be beneficial.

#### Intraplant Variation in Carbon and Nitrogen Isotopic Compositions

Plant nitrogen isotopic composition did not systematically vary between different tissues sampled. On the basis of hydroponic studies, significant intraplant variation (between roots and shoots) is only expected when plants are fed with NO_3_^−^ as the N source [166]. Additionally, plant δ^15^N may vary considerably among tissues due to biochemical processes associated with growth and senescence over time [143], [211]–[213]. The lack of any clear pattern of intraplant variation in δ^15^N likely relates to a number of factors, including: variable reliance on different N sources (nitrate, ammonium, organic N) by different plant taxa and between sampling locations, differences in plant life cycles between different taxa, and spatial variation in the influence of environmental factors on the isotopic composition of source N.

Foliar tissues tended to be more depleted of ^13^C than other tissues (Figure 9). The magnitude of this difference was typically ≤1 ‰, but was absent for C_4_ plants. This fits with previously described data for other plants. The small difference in δ^13^C among plant tissues is not likely to be significant with respect to the interpretation of isotopic data in the context of paleodietary studies.

#### Geographic Variation in Carbon and Nitrogen Isotopic Compositions

There were strong relationships between sampling site and foliar carbon and nitrogen isotopic compositions. Foliar δ^15^N was negatively correlated with altitude (Figure 10a) and mean annual precipitation (Figure 11a), although based on the large number of studies finding a strong relationship between rainfall amount and soil, plant, and animal δ^15^N [12], [15], [85]–[87], [172], [176]–[179], this relationship is likely driven by rainfall. This suggests that arid sites are characterized by a fairly open nitrogen cycle, as described in previous studies [179]. It is unclear to what extent these processes would act on agricultural plants growing in relatively arid versus wet sites. Even on the hyper-arid coast where rainfall is negligible, agriculture is made possible by substantial irrigation networks. Hence, water availability in agricultural contexts is markedly higher than in non-irrigated areas. Agricultural products grown in coastal regions of the central Andes may therefore not be characterized by higher δ^15^N values relative to those growing at wetter, higher altitude sites. For instance, maize grown as part of a controlled experiment (no fertilization) located ∼6 km from the coast, had grain δ^15^N values of 6.3±0.3 ‰ [147], comparable to results for maize growing at higher altitudes in this study (6.4±2.2 ‰). Aside from issues of irrigation, agricultural plants analyzed in this study were sampled along a relatively limited altitudinal transect (2233 to 3588 masl) where effects on tissue δ^15^N values would be expected to be more limited (Figure 10a).

The positive relationship found between rainfall and foliar δ^13^C in C_3_ plants contrasts with most other studies, which have typically found a negative relationship between rainfall and foliar δ^13^C. The majority of these studies, however, sampled plants along a large rainfall gradient (>1,000 mm), but with little difference in elevation between sites. Conversely, we sampled along a more restricted rainfall gradient (∼700 mm), but a very large altitudinal gradient (∼4,000 m). Increased altitude and increased rainfall have opposing effects on foliar δ^13^C values, and the results of this study suggest the predominance of altitudinal effects on foliar carbon isotopic compositions in northern Peru. A similar pattern was observed along a comparable altitudinal gradient in northern Chile (Figure 12). This pattern is most likely related to high carboxylation rates relative to stomatal conductance at high altitudes resulting in lower ^13^C discrimination. Such effects should be equally apparent in cultivated plants, although they were not observed in this study because of the limited altitudinal range from which cultigens were sampled (Table 2).

Variation in plant isotopic compositions along environmental gradients is particularly important with respect to the reconstruction of the diet of humans and animals using isotopic data. While the majority of wild plants analyzed in this study would not have been consumed by humans, the results are very relevant to the reconstruction of animal management practices. There is considerable debate in the Andean region with respect to the herding practices of South American camelids (llama and alpaca), and whether or not animals recovered from coastal sites were raised locally, or imported from elsewhere [214]. The results of this study suggest that animals feeding on wild plants at drier, low altitude sites would be characterized by higher tissue δ^15^N values than animals feeding on wild plants at wetter, high altitude sites. The magnitude of this difference could easily be 4 to 6 ‰, although the consumption of agricultural plants dependent on irrigation at lower altitudes could serve to obscure this difference (as discussed above).

The potential consequences of altitudinal variation in plant δ^13^C values are more difficult to evaluate. While the positive linear relationship between altitude and foliar δ^13^C is strong, the relative distribution of C_3_ and C_4_ plants would serve to counter these effects. Because there will be proportionately more C_4_ plants at dry, low altitude sites relative to moister, high altitude sites, the average δ^13^C value of available forage would still be higher at low altitude sites. Thus, markedly higher δ^13^C and δ^15^N values observed in some camelids from low altitude sites [38], [215] can be satisfactorily explained by the consumption of local terrestrial vegetation.

### Marine Plants

Marine algae are known to have been an important dietary resource for many groups of people in the coastal regions of Peru and Chile [216], but the lack of preservation of marine algae in all but the most exceptional archaeological contexts makes evaluating the potential importance of marine algae in the diet extremely difficult. Marine plants were characterized by δ^13^C values intermediate between C_3_ and C_4_ plants, with δ^15^N values comparable to terrestrial plants growing on the coast. DeNiro [215] has suggested that consumption of marine algae may have been responsible for relatively high δ^13^C and δ^15^N values in coastal Peruvian camelids. While the number of macroalgal species sampled in this study is not extensive, the isotopic data presented here are not consistent with this explanation. With the exception of instances in which marine plants grow in areas of exceptionally high influence of marine bird and/or mammalian excreta [217], there is no reason to expect marine algal δ^15^N values to be higher than the δ^15^N values of plants growing along the arid coast of Peru.

## Conclusions

Maize from the study area has a mean δ^13^C value of −11.8±0.4‰, which suggests that a δ^13^C value (adjusted for the Suess Effect) of ca. −10.3 ‰ would be appropriate for paleodietary models in the region. Leguminous cultigens were characterized by significantly lower δ^15^N values and higher N contents than non-leguminous cultigens; this distinction was not as clear for wild legumes. Marine plants were characterized by δ^13^C values intermediate between wild terrestrial C_3_ and C_4_ vegetation and δ^15^N values that were very similar to terrestrial plants growing at low altitudes. C_4_ plants were generally more abundant at lower altitude sites. Carbon and nitrogen isotopic compositions of wild plants were strongly influenced by local environmental factors. Foliar δ^13^C was positively correlated with altitude and negatively correlated with mean annual precipitation. Foliar δ^15^N was negatively correlated with altitude and mean annual precipitation.

While the last twenty years have seen a proliferation of studies utilizing the isotopic analysis of archaeological materials for the purpose of reconstructing diet, the development of isotopic baselines for interpreting such data has lagged behind these investigations. This hampers our ability to realize the full potential of isotopic data. This study begins to fill part of that gap by providing an initial understanding of the baseline isotopic variation in plants from northern Peru. Further studies of this nature are required to better understand baseline isotopic variation in other regions.

## Supporting Information

Dataset S1 **Sampling site locations for wild and market plants.** This.kmz file can be executed in Google Earth (http://www.earth.google.com)
(KML)

Table S1 **Isotopic and elemental data for all cultigens analyzed.**
(XLS)
